# Chemotherapy‐Induced Nausea and Vomiting in Early Breast Cancer Patients Receiving Adjuvant Chemotherapy With Fluorouracil, Epirubicin, Cyclophosphamide Followed by Docetaxel Versus an Anthracycline‐Free Regimen With Docetaxel, Cyclophosphamide—Results From a Randomized Clinical Trial

**DOI:** 10.1002/ijc.70528

**Published:** 2026-04-29

**Authors:** Manuel Hörner, Henning Schäffler, Lothar Häberle, Chloë Goossens, Kerstin Pfister, Elena Leinert, Kristina Veselinovic, Sara Y. Brucker, Uwe Köhler, Georg Heinrich, Andreas Schneeweiss, Matthias W. Beckmann, Peter A. Fasching, Wolfgang Janni, Brigitte Rack, Sabine Heublein, Philipp Ziegler

**Affiliations:** ^1^ Department of Gynecology and Obstetrics University Hospital Erlangen, Comprehensive Cancer Center Erlangen‐EMN (CCC ER‐EMN), Friedrich‐Alexander‐Universität Erlangen‐Nürnberg (FAU) Erlangen Germany; ^2^ Department of Gynecology and Obstetrics Ulm University Hospital Ulm Germany; ^3^ Biostatistics Unit, Department of Gynecology and Obstetrics Universitätsklinikum Erlangen Erlangen Germany; ^4^ Department of Women's Health Tuebingen University Tuebingen Germany; ^5^ Department of Gynecology and Obstetrics Klinikum St. Georg Leipzig Germany; ^6^ Schwerpunktpraxis der Gynäkologie und Onkologie Fürstenwalde, Klinikum Offenbach Fürstenwalde Germany; ^7^ National Center for Tumor Diseases University Hospital and German Cancer Research Center Heidelberg Heidelberg Germany; ^8^ German Cancer Research Center (DKFZ) Heidelberg Germany

**Keywords:** chemotherapy, chemotherapy‐induced nausea and vomiting (CINV), early breast cancer, randomized clinical trial

## Abstract

Chemotherapy‐induced nausea and vomiting (CINV) remains an important side effect despite new antiemetic drugs. This study tried to understand the occurrence of CINV in patients receiving two different chemotherapy regimens. As part of the randomized controlled clinical trial SUCCESS C (NCT00847444), 1582 of the 3463 patients completed CINV diaries. Patients were randomized to receive either chemotherapy with FEC (5‐fluorouracil, epirubicin, cyclophosphamide followed by docetaxel) or TC (docetaxel, cyclophosphamide). CINV was evaluated hourly using a specially designed questionnaire. Endpoints of the study were complete response (no emesis) and total control (no nausea and no emesis) and were assessed with Kaplan–Meier curves and Cox regression analyses over three chemotherapy cycles. Eight hundred fourteen patients received FEC and 768 received TC; patients and tumor characteristics were similar in both groups. Patients receiving FEC had significantly more nausea and vomiting, with the main difference in the first 12 h. In the first cycle, the 0–12‐h nausea/emesis‐free rates were 70%/41% for FEC and 91/76% for TC. By 24 h after chemotherapy, the rates were 65%/33% (FEC) and 85%/60% (TC). The differences were similar in cycles 2 and 3. The detailed analysis of CINV in the study is unique and paves the way for modern CINV analysis of new therapeutics such as antibody‐drug conjugates.

## What's New?

Chemotherapy‐induced nausea and vomiting (CINV) remains a significant clinical problem despite modern antiemetics. In particular, antiemetic strategies for emerging cancer therapies lack detailed evaluation. This randomized trial analyzed hourly CINV data for patients with early breast cancer who received either docetaxel plus cyclophosphamide (TC) or fluorouracil, epirubicin, and cyclophosphamide (FEC) followed by docetaxel. Significantly higher rates of nausea and vomiting were observed with FEC, particularly within the initial 12 hours post‐treatment. Nausea and emesis control improved in subsequent treatment cycles. The findings highlight regimen‐specific differences and underscore the value of detailed temporal monitoring for improving CINV management in cancer patients.

## Graphical abstract

Chemotherapy‐induced nausea and vomiting (CINV) remains a significant clinical problem despite modern antiemetics. In particular, antiemetic strategies for emerging cancer therapies lack detailed evaluation. This randomized trial analyzed hourly CINV data for patients with early breast cancer who received either docetaxel plus cyclophosphamide (TC) or fluorouracil, epirubicin, and cyclophosphamide (FEC) followed by docetaxel. Significantly higher rates of nausea and vomiting were observed with FEC, particularly within the initial 12 h post‐treatment. Nausea and emesis control improved in subsequent treatment cycles. The findings highlight regimen‐specific differences and underscore the value of detailed temporal monitoring for improving CINV management in cancer patients.

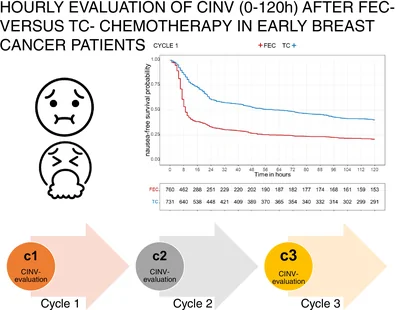

**Abbreviations**
5‐HT3‐R: 5‐Hydroxytryptophan3 receptor
5‐HTP: 5‐Hydroxytryptophan
ADC: antibody‐drug conjugate
BMI: body mass index
CI: confidence interval
CINV: chemotherapy‐induced nausea and vomiting
CR: complete response
ECOG: eastern cooperative oncology group performance status scale
FEC: fluorouracil, epirubicin, cyclophosphamide
HER2: human epidermal growth factor receptor 2
HR: hazard ratio
MCP: metoclopramide
NK‐1: neurokinin‐1
SUCCESS C: Simultaneous Study of Docetaxel‐based Anthracycline‐free adjuvant Treatment Evaluation, plus Lifestyle Intervention Strategies
TC: docetaxel, cyclophosphamide
TNBC: triple negative breast cancer
VC: vomiting center

## 1 Introduction

Chemotherapy‐induced nausea and vomiting (CINV) is a severe and frequent side effect affecting patients treated with cytotoxic agents. Hesketh et al. proposed a five‐level classification system for the emetogenic potential of chemotherapeutic regimens, which was later simplified to a four‐level system at the 2004 Perugia Consensus conference, categorizing regimens as minimally, low, moderately, and highly emetogenic [1, 2]. Each regimen used in this analysis was assigned to one of the former categories. Despite the development of highly potent antiemetic therapies, CINV remains a relevant issue, leading to a reduction in quality of life, treatment delays, dose reductions, and therapy discontinuation [3]. With the introduction of antibody‐drug conjugates (ADCs), dealing with CINV regains importance [4].

Different stages of CINV have been described: acute (occurring within the first 24 h), delayed (occurring between day 2 and day 5), anticipatory (occurring before chemotherapy as a conditioned response due to previously experienced CINV), breakthrough (occurring during the first 5 days after chemotherapy despite antiemetic therapy), and refractory CINV (occurring in later cycles as a result of ineffective antiemetic therapy in prior cycles). Importantly, these different stages of CINV all require different therapeutic strategies [3].

The etiology of acute versus delayed CINV also differs. Cytostatic agents activate the vomiting center (VC, a network of neurons in the medulla oblongata in the brain stem) leading to acute CINV. In the periphery, afferent vagal neurons in the gastrointestinal tract are activated through different pathways and neurotransmitters such as 5‐hydroxytryptophan (5‐HTP), neurokinin‐1 (NK‐1) and Cholezystokinin [3]. The afferent fibers of the neuron end in the nucleus tractus solitarius and the area postrema activating the VC. Those two mechanisms lead to the acute CINV. Other substances involved are substance P, dopamine, and opioids acting as agonists or antagonists directly in the area postrema due to its lack of the blood–brain barrier. Delayed CINV is partly explained by the build‐up of free oxygen radicals after cytotoxic events and cell death, and by a prolonged activation of 5‐HT3 receptors in the gastrointestinal tract. In addition, other factors such as psychological distress, direct effects of the primary disease, delayed gastric emptying, and the effects of other non‐chemotherapeutic medications play an important role [5].

Several risk factors for the development of CINV have been described, including the type of chemotherapy, previous history of CINV, first cycle, younger age, poor sleep prior to chemotherapy, female gender, alcohol use, antiemetic use, previous pregnancy‐induced nausea and vomiting, and history of motion sickness [5]. The management of CINV is well established with the use of prophylactic antiemetic regimens that depend on the emetic potential of the corresponding chemotherapy. These regimens include one to three antiemetic drugs given on the day of chemotherapy to prevent acute CINV and may be given up to 3–4 days after chemotherapy to prevent delayed CINV.

The current landscape on CINV research focuses on phase II and III pharmacological intervention trials. The most recent antiemetic drug, the NK1 receptor antagonist rolapitant, was launched in 2015, suggesting that the development of antiemetic drugs is not a high priority in the breast cancer research community [6]. Furthermore, studies prospectively collecting high‐resolution CINV data in large patient cohorts of more than 1000 patients are rare [7]. Dranitsaris et al. included 1198 patients with various cancers in the analysis for the development of a CINV risk prediction tool [8]. Tamura et al. evaluated CINV in two different chemotherapy regimens in a prospective registry study involving 1910 patients [9]. Here, data was collected on a daily basis for the first 7 days after chemotherapy. Roscoe et al. prospectively evaluated a high‐risk regimen of CINV four times daily using a Likert scale ranging from 0 to 7, thus also neglecting high‐resolution CINV evaluation [10]. Risk factors found in these studies were female gender, alcohol consumption, expectancy of CINV, history of nausea and vomiting, younger age, history of morning sickness and anxiety, with nicotine use possibly being a protective factor [11].

Notably, these studies evaluated older anticancer treatments, and prospective trials reliably evaluating antiemetic approaches for newer treatments such as ADCs are still lacking. The evaluation of CINV at high temporal resolution also requires further research. Therefore, this analysis aims to provide a detailed characterization of CINV using established endpoints based on high‐resolution data collection under antiemetic regimens still in use today.

## 2 Materials and Methods

### 2.1 SUCCESS C Trial

The SUCCESS C trial (NCT00847444), enrolled patients with human epidermal growth factor receptor 2 (HER2)‐negative early breast cancer with either evidence of positive axillary lymph nodes (pN1) or high‐risk node‐negative disease (at least pT > 1 or G3 or age < 36 years or triple negative breast cancer (TNBC)) after primary R0 surgery with an indication for adjuvant chemotherapy [12]. Between February 2009 and August 2011, 3642 pre‐ and postmenopausal women with newly diagnosed HER2‐negative intermediate‐risk to high‐risk breast cancer were enrolled in 231 study centers across Germany [13]. In this open label trial, patients were randomized to receive either 3 cycles of 5‐fluoruoracil (500 mg/m^2^ i.v.)/epirubicin (100 mg/m^2^ i.v)/cyclophosphamide (500 mg/m^2^ i.v.) (FEC) every 21 days (q21d) followed by 3 cycles of docetaxel (100 mg/m^2^) q21d or 6 cycles of docetaxel (75 mg/m^2^ i.v.)/cyclophosphamide (600 mg/m^2^ i.v.) (TC) q21d. After adjuvant chemotherapy, a second randomization took place for either phone‐based individual lifestyle intervention or general lifestyle recommendations in the control arm. Neither randomization was blinded.

### 2.2 Data Collection

During adjuvant chemotherapy, CINV was evaluated using a study‐specific emesis diary during the first 5 days after receiving chemotherapy that included hourly evaluation of nausea, retching, vomiting, exact intake of antiemetic medication, eating and drinking habits. The diary was explained in detail at the start of chemotherapy, handed out on the first day of each cycle and returned on the first day of the following cycle or at the first follow‐up visit for the last cycle.

As described in the SUCCESS C study protocol, the recommended antiemetic prophylaxis in the FEC arm (cycle 1–3) consisted of a NK1 receptor antagonist according to the summary of product characteristics (e.g., aprepitant (125 mg on day 1 followed by 80 mg on days 2 and 3)), dexamethasone (12 mg i.v.) and ondansetron (8 mg twice daily p.o. or equivalent) on day 1 (day of chemotherapy), a NK‐1‐receptor antagonist on day 2–4, and dexamethasone (8 mg p.o. or i.v.) on day 2–3. In the TC arm, recommended antiemetic prophylaxis was dexamethasone (8 mg p.o.) on the day before chemotherapy, dexamethasone (8 mg i.v. or p.o.) on the day of chemotherapy and the day after, and ondansetron (8 mg i.v.) on the day of chemotherapy.

### 2.3 Data Preparation for Analysis

#### 2.3.1 Emesis Diary

The emesis patient diary consisted of 18 questions on health habits and living conditions and a recurring set of two tables, and was handed out for six consecutive days: from the day of chemotherapy until the fifth day after chemotherapy. In the first table, nausea, retching, vomiting, and application of antiemetic medications, eating and drinking was documented hourly. In the second table, the type of applied antiemetic medications was documented daily. Nausea was documented in three levels: mild, moderate, or severe.

Data from the emesis patient diaries were manually transferred to an excel database. The weekly consumption of alcoholic beverages (never, 1–2 times weekly, 3–6 times weekly, daily) and the first occurrence of any type of event in the first table were identified. Medication documentation in the second table was classified into categories (aprepitant, dexamethasone, ondansetron or equivalent, metoclopramide (MCP), dimenhydrinate).

#### 2.3.2 Additional Characteristics

From case report forms, the continuous variables age and body mass index (BMI) were extracted. Furthermore, the ordinal variables eastern cooperative oncology group performance status scale (ECOG) (0, 1, 2+), pathological tumor status (pT1, pT2, pT3+), pathological node status (pN0, pN+) and smoking (smoker, non‐smoker) were captured. For smoking, the additional category occasional smoker was subordinated to smoker, as the self‐attribution of occasional smoking is often ambivalent [14]. For chemotherapy cycles two and three, the occurrence of endpoint events (see below) for former cycles was computed as a binary variable (yes, no).

#### 2.3.3 Endpoints

A measure for nonoccurrence of CINV is complete response (CR) [11, 15]. CR is the combined endpoint of no emetic event, defined as vomiting or retching and no need for rescue antiemetic therapy (such as MCP). Complete control is the combined endpoint of no emetic event, no retching, no need for rescue antiemetic therapy, and no significant nausea (manageable and, if at all, only minimally affects daily activities). Total control is the combined endpoint of no emetic event, no retching, no need for rescue antiemetic therapy, and no nausea at all [15].

Furthermore, an emetic episode was defined as the occurrence of retching, vomiting, or the application of rescue medication. A nausea episode was defined as the occurrence of an emetic episode or of nausea. CR time was defined as the duration in hours from receiving chemotherapy to the earliest occurring emetic episode. Total control time was defined as the duration in hours from receiving chemotherapy to the earliest occurring nausea episode. In survival analysis terms, one could refer to CR as emesis‐free survival and to total control as nausea‐free survival. Observation time was censored at 120 h.

Emesis‐free rate and nausea‐free rate were evaluated for the first 12, 24, 48, and 120 h. Of note, the nausea‐free rate for the first 24 h is equivalent to the acute total control rate (24 h); the nausea‐free rate for the first 120 h is equivalent to the overall total control rate (120 h).

### 2.4 Statistical Analysis

The primary objective was to investigate whether the chemotherapy regimen (TC vs. FEC) affects CR and/or total control in addition to other well‐known prognostic factors. To check for differences in CR and/or total control, the first three chemotherapy cycles were considered. For this purpose, for each of the three chemotherapy cycles, a multiple Cox regression model was fitted with the outcome CR or total control, and the predictors therapy type (categorial; TC, FEC), patient age (continuous), smoking (categorial; non‐smoker, smoker), alcohol per week (categorial; never, 1–2 times weekly, 3–6 times weekly, daily), BMI (continuous), pre‐cycle event (categorial; yes, no) (the full model). Additionally, for every chemotherapy cycle, another multiple Cox regression model with the same predictors as the full model, except therapy type, was fitted with outcome CR, respectively total control (base model).

For every cycle, the full model and the base model were compared using the likelihood ratio test. Bonferroni corrections for multiple testing were applied. For each outcome, the *p* values of the three statistical tests were determined. If at least one of these *p* values was below the significance level 0.05/3, a significant relation was found. Chemotherapy regimen would then be a relevant predictor for CR, respectively total control.

Patients with missing outcome values were excluded. Missing data on smoking was imputed as the most frequent level, which was non‐smoker. As the other predictors were complete or had no dominant level, no further imputation was performed.

If a predictor (except the predictor of interest) violated the proportional hazard assumption, it was considered as a stratification factor within the Cox regression model. For all models, it was necessary to consider age as a stratification factor (see stratification levels in Table 1). For CR models, it was necessary to consider smoking as a stratification factor in cycle 1; a pre‐cycle event was considered as a stratification factor in cycles 2 and 3.

**TABLE 1:** Patient characteristics for both chemotherapy schemata and whole population (1582 patients), showing mean and standard deviation or frequency and percentage.

Characteristic		Therapy type		Total
		FEC (*N* = 814)	TC (*N* = 768)	
Age	Mean (sd)	54.6 (10.1)	55.2 (10.1)	54.9 (10.1)
	Age < 50	258 (31.7%)	247 (32.2%)	505 (31.9%)
	50 ≤ Age < 65	408 (50.1%)	368 (47.9%)	776 (49.1%)
	Age ≥ 65	148 (18.2%)	153 (19.9%)	301 (19.0%)
Alcohol per week	Never	342 (44.8%)	315 (44.4%)	657 (44.6%)
	1–2×	339 (44.4%)	306 (43.2%)	645 (43.8%)
	3–6×	61 (8.0%)	71 (10.0%)	132 (9.0%)
	Daily	21 (2.8%)	17 (2.4%)	38 (2.6%)
	Missing	51	59	110
Smoking	Non‐smoker	665 (89.6%)	638 (89.4%)	1303 (89.5%)
	Smoker	77 (10.4%)	76 (10.6%)	153 (10.5%)
	Missing	72	54	126
Body mass index	Mean (sd)	26.4 (5.2)	26.7 (5.2)	26.5 (5.2)
Pre‐cycle emetic episode	No	380 (47.7%)	482 (64.4%)	862 (55.8%)
	Yes	417 (52.3%)	266 (35.6%)	683 (44.2%)
	Missing	17	20	37
Pre‐cycle nausea episode	No	129 (16.2%)	243 (32.5%)	372 (24.1%)
	Yes	668 (83.8%)	505 (67.5%)	1173 (75.9%)
	Missing	17	20	37
Prophylactic antiemetic medication	Aprepitant	198 (24.3%)	62 (8.1%)	260 (16.4%)
	Dexamethasone	220 (27.0%)	232 (30.2%)	452 (28.6%)
	Ondansentron or eq.	167 (20.5%)	100 (13.0%)	267 (16.9%)
Antiemetic rescue medication	MCP	278 (34.2%)	208 (27.1%)	486 (30.7%)
	Dimenhydrinat	52 (6.4%)	14 (1.8%)	66 (4.2%)
ECOG	0	522 (71.7%)	499 (71.3%)	1021 (71.5%)
	1	199 (27.3%)	193 (27.6%)	392 (27.5%)
	2+	7 (1.0%)	8 (1.1%)	15 (1.1%)
	Missing	86	68	154
Molecular subtype	Luminal A	440 (54.1%)	408 (53.1%)	848 (53.6%)
	Luminal B	172 (21.1%)	172 (22.4%)	344 (21.7%)
	TNBC	202 (24.8%)	188 (24.5%)	390 (24.7%)
Pathological tumor status	pT1	358 (44.0%)	337 (43.9%)	695 (43.9%)
	pT2	400 (49.1%)	385 (50.1%)	785 (49.6%)
	pT3+	56 (6.9%)	46 (6.0%)	102 (6.4%)
Pathological node status	pN0	347 (42.6%)	310 (40.4%)	657 (41.5%)
	pN+	467 (57.4%)	458 (59.6%)	925 (58.5%)

Abbreviations: ECOG, Eastern Cooperative Oncology Group performance status scale; FEC, fluoracil, epirubicin, cyclophosphamide; sd, standard deviation; TC docetaxel, cyclophsophamid; TNBC, triple negative breast cancer.

The proportional hazards assumptions were not met for chemotherapy regimen (which is the predictor of interest) on emesis‐free survival and nausea‐free survival. Thus, the overall observation period was divided in three parts, each of which fulfilled the proportional hazards assumptions: 0–12 h (acute phase I), 12–24 h (acute phase II), and 24–120 h (delayed phase) after drug administration.

Adjusted overall hazard ratios (HRs) for chemotherapy regimen were estimated for each of the three observation periods. As a sensitivity analysis, unadjusted HRs were estimated for CR and total control, using a Cox regression model with only the chemotherapy regimen as the predictor.

Unadjusted nausea‐free rates and emesis‐free rates with 95% confidence intervals (CIs) and median nausea‐free times were estimated using the Kaplan–Meier product limit method. The 95% CI of median nausea‐free times was computed using the method of Brookmeyer and Crowley (1982). Emesis‐free rates did not fall below 50%, thus median emesis‐free times could not be estimated.

Some cycles were documented but lacked recorded times of chemotherapy application. If the patient had documented times in other chemotherapy cycles, the time was imputed by the median of these application times, since the timing of chemotherapy administrations between cycles was strongly correlated within the same patient.

Statistical tests were two‐sided with a 5% significance level. Data was prepared with T‐SQL on Microsoft SQL Server Enterprise (64‐bit, Version 14.0.3485.1). Calculations were performed with R statistical computing (version 4.2.3, Shortstop Beagle) within the RStudio environment.

## 3 Results

**FIGURE 1:**
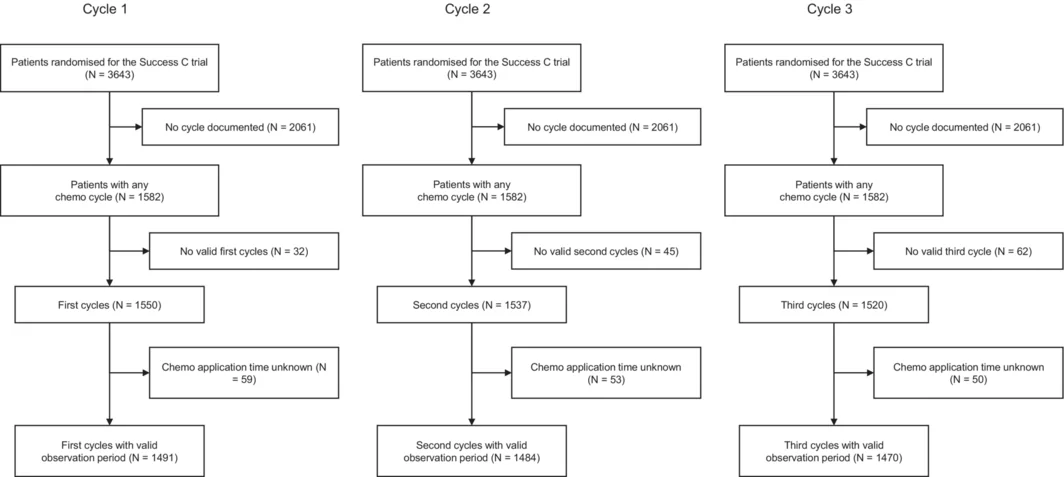
Patient flow chart showing reasons for patient exclusion in each of the three cycles.

**FIGURE 2:**
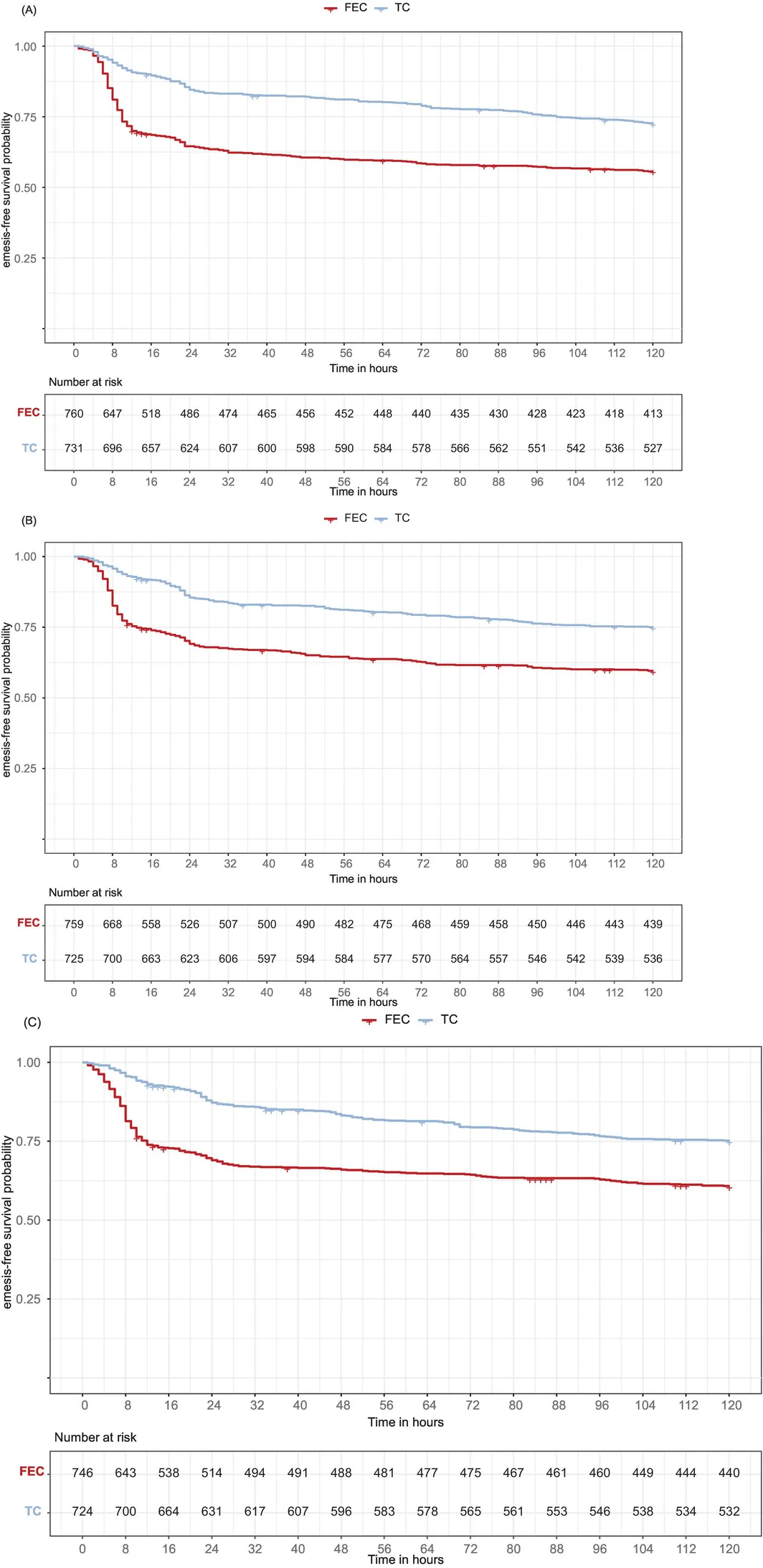
Complete response (emesis‐free survival) described via Kaplan Meier curves between the FEC and the TC group. (A) shows cycle 1, (B) cycle 2, and (C) cycle 3. Patients at risk are displayed below each curve. (A) Complete response (emesis‐free survival) described via Kaplan Meier curves between the FEC and the TC for cycle 1. (B) Complete response (emesis‐free survival) described via Kaplan Meier curves between the FEC and the TC for cycle 2. (C) Complete response (emesis‐free survival) described via Kaplan Meier curves between the FEC and the TC for cycle 3.

Of the 3463 patients included in the SUCCESS C trial, 2061 patients had to be excluded due to missing documented chemotherapy cycles, resulting in 1582 patients for this analysis. Further information on patient exclusions is provided in Figure 1. Of the 814 patients that received FEC, the average age was 54.6 years and the average BMI was 26.4 (Table 1). Furthermore, 44.8% of patients never drank alcohol and 89.6% were non‐smokers. The majority of patients had an ECOG status of 0 (71.7%) and had a luminal A‐like tumor (54.1%). The most common pathological tumor stage was pT2 (49.1%) and 57.4% of the patients had positive lymph nodes. Patients that received TC (*N* = 768) were on average 55.2 years old, and had a BMI of 26.7. In addition, 44.4% of patients never drank alcohol and 89.4% were non‐smokers. 71.3% of patients had an ECOG of 0, 53.1% had a luminal A‐like tumor and most of the patients also had a T2 pathological tumor stage (50.1%) and positive lymph nodes (59.6%). Overall, patient characteristics were comparable between chemotherapy groups, for more details, see Table 1. CR varied between FEC and TC groups. Patients receiving FEC had more emetic events (336 in cycle 1, 305 in cycle 2 and 291 in cycle 3) than patients in the TC group (200 in cycle 1, 180 in cycle 2 and 178 in cycle 3), see Table 2. Most of the emetic events occurred during the first 24 h after receiving chemotherapy, with the majority even in the first 12 h, see Table 2 and Figure 2. For cycle 1, the median emesis‐free rate for FEC is 70% in the first 12 h, 65% in the first 24 h and 56% in the first 120 h (Table 2). For TC in cycle 1, the median emesis‐free rate is 91% in the first 12 h and 73% in the 120 h following chemotherapy, resulting in a significant difference between the two groups. The overall emesis‐free rate (120 h) increased from 56% for FEC in cycle 1 to 61% in cycle 3 and for TC from 73% in cycle 1 to 75% in cycle 3. For more details, see Table 2.

**TABLE 2:** Median emesis‐free rates, Median nausea‐free times and nausea‐free rates relative to chemotherapy regimen and cycle^a^.

Event type	Chemo‐therapy	Chemo‐cycle	Patients	Events	Median event‐free time in hours (95% CI)	12‐h event‐free rate (95% CI)	24‐h event‐free rate (95% CI)	48‐h event‐free rate (95% CI)	120‐h event‐free rate (95% CI)
Emesis	FEC	1	760	336	^a^	0.70 (0.67, 0.73)	0.65 (0.61, 0.68)	0.61 (0.57, 0.64)	0.56 (0.52, 0.59)
		2	759	305	^a^	0.75 (0.72, 0.78)	0.69 (0.66, 0.72)	0.65 (0.62, 0.69)	0.60 (0.56, 0.63)
		3	746	291	^a^	0.74 (0.71, 0.77)	0.69 (0.66, 0.72)	0.66 (0.63, 0.69)	0.61 (0.57, 0.64)
	TC	1	731	200	^a^	0.91 (0.89, 0.93)	0.85 (0.82, 0.87)	0.82 (0.79, 0.85)	0.73 (0.69, 0.76)
		2	725	180	^a^	0.93 (0.91, 0.95)	0.85 (0.83, 0.88)	0.83 (0.80, 0.85)	0.75 (0.72, 0.78)
		3	724	178	^a^	0.93 (0.91, 0.95)	0.87 (0.85, 0.90)	0.83 (0.81, 0.86)	0.75 (0.72, 0.78)
Nausea	FEC	1	760	599	9.0 (8.0, 10.0)	0.41 (0.38, 0.45)	0.33 (0.30, 0.37)	0.27 (0.24, 0.30)	0.21 (0.18, 0.24)
		2	759	561	10.0 (9.0, 12.0)	0.46 (0.43, 0.50)	0.36 (0.33, 0.40)	0.31 (0.28, 0.34)	0.26 (0.23, 0.29)
		3	746	523	10.5 (9.0, 14.0)	0.47 (0.44, 0.51)	0.40 (0.37, 0.44)	0.36 (0.33, 0.40)	0.30 (0.26, 0.33)
	TC	1	731	436	64.0 (47.0, 82.0)	0.76 (0.73, 0.80)	0.60 (0.57, 0.64)	0.53 (0.49, 0.57)	0.40 (0.37, 0.44)
		2	725	377	88.0 (71.0, NA)	0.83 (0.80, 0.86)	0.67 (0.64, 0.70)	0.60 (0.57, 0.64)	0.48 (0.44, 0.52)
		3	724	342	NA (97.0, NA)	0.82 (0.80, 0.85)	0.72 (0.69, 0.75)	0.62 (0.59, 0.66)	0.52 (0.49, 0.56)

*Note:* Emesis‐free rates could also be referred to as complete response rates. 24‐h emesis‐free rate is equivalent to acute complete response rate, 120‐h emesis‐free rate to overall complete response rate. Nausea‐free rates could also be referred to as total control rates, median nausea‐free time as median total control time. 24‐h nausea‐free rate is equivalent to acute total control rate, 120‐h nausea‐free rate to overall total control rate.

Abbreviation: CI, confidence interval.

^a^ Median emesis‐free time was not reached within 120 h observation period.

**FIGURE 3:**
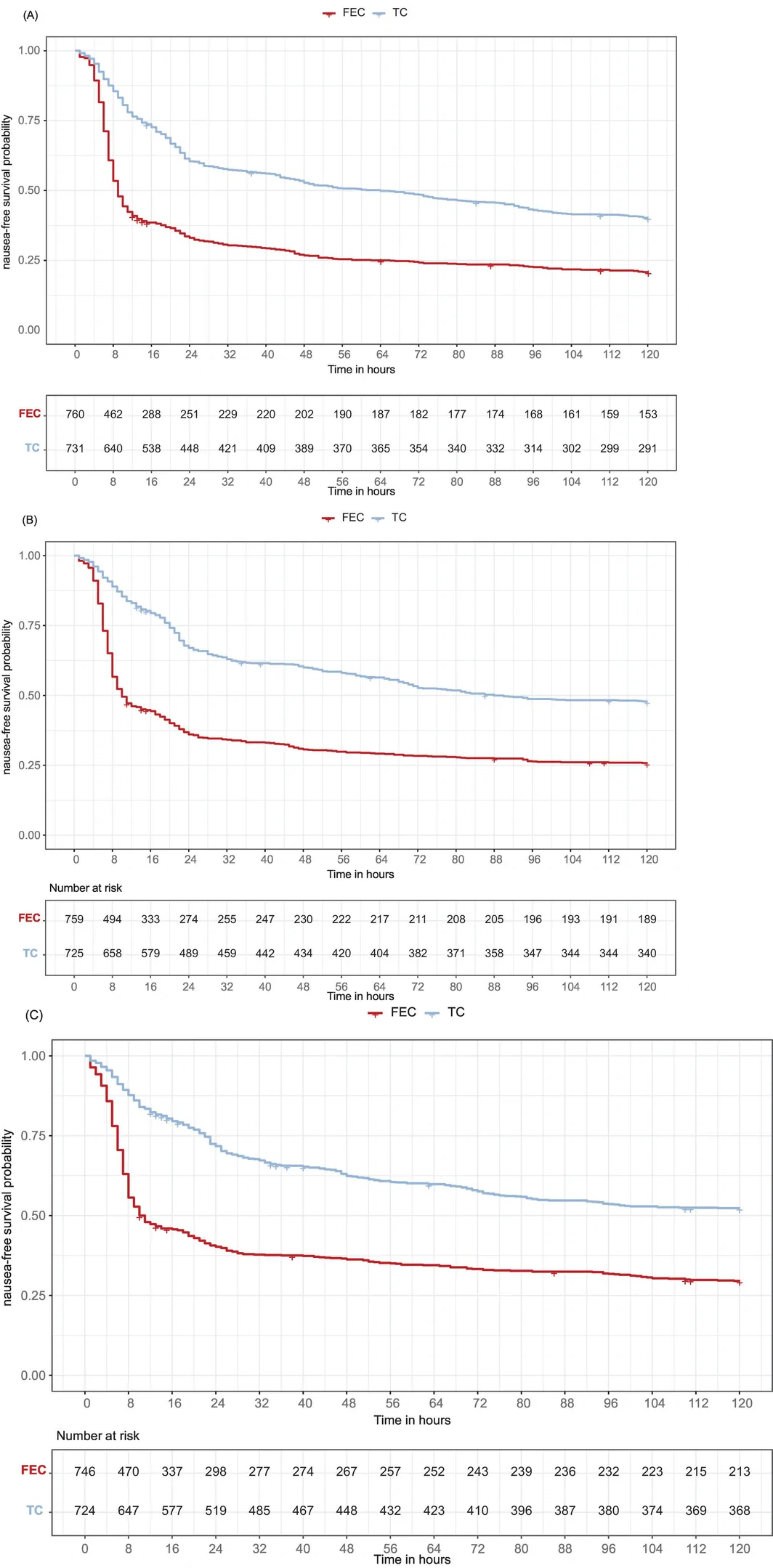
Total control (nausea‐free survival) described via Kaplan Meier curves between the FEC and the TC group. (A) shows cycle 1, (B) cycle 2, and (C) cycle 3. Patients at risk are displayed below each curve. (A) Total control (nausea‐free survival) described via Kaplan Meier curves between the FEC and the TC group for cycle 1. (B) Total control (nausea‐free survival) described via Kaplan Meier curves between the FEC and the TC group for cycle 2. (C) Total control (nausea‐free survival) described via Kaplan Meier curves between the FEC and the TC group for cycle 3.

Total control differed between both groups as well. With consideration not only of emetic events but also nausea, the difference between groups was even more profound. Patients receiving FEC had more nausea events (599 in cycle 1, 561 in cycle 2 and 523 in cycle 3) than patients in the TC group (436 in cycle 1, 377 in cycle 2 and 342 in cycle 3). Nausea‐free rates were lower than emesis‐free rates in both groups. As with CR, most events occurred in the acute phase, especially within the first 12 h, with fewer events taking place between 24 and 120 h (delayed) after receiving chemotherapy, see Table 2 and Figure 3. For FEC in cycle one, the 12‐h total control rate was 41%, 33% for the first 24 h and 21% for the first 120 h (Table 2). For TC in cycle 1, the 24‐h total control rate was 60% and the 120‐h (overall) total control rate was 30%. The overall nausea‐free rates increased from 21% for FEC in cycle 1 to 30% in cycle 3 and for TC from 40% in cycle 1 to 52% in cycle 3. For further details, see Table 2.

Chemotherapy type (FEC vs. TC) had an impact on total control (smallest *p* value over all three cycles < 0.00001, likelihood ratio test) and on CR (smallest *p* value over all three cycles < 0.00001, likelihood ratio test).

Adjusted and unadjusted HRs for FEC versus TC apparently differed in the time frame 0–12 h after receiving chemotherapy. The adjusted HRs for CR were 3.54 (95% CI; 2.67, 4.68), 2.61 (95% CI; 1.89, 3.60), and 3.12 (95% CI; 2.26, 4.32) for cycles 1, 2, and 3 respectively. For total control, adjusted HRs were 3.31 (95% CI; 2.75, 3.97), 3.35 (95% CI; 2.72, 4.14), and 3.18 (95% CI; 2.58, 3.92) for cycles 1, 2, and 3. The HRs for 12–24 h and 24–120 h after chemotherapy showed no significant differences between the two chemo regimens with HRs around 1 for all 3 cycles for both outcomes, CR and total control. For further details, see Table 3.

**TABLE 3:** Cox regression analyses, showing unadjusted and adjusted hazard ratios for FEC versus. TC for 0 h–12 h, for 12 h–24 h and for 24 h–120 h after chemotherapy application. [h hours].

Outcome	Cycle	Therapies	0 h–12 h after chemotherapy		12 h–24 h after chemotherapy		24 h–120 h after chemotherapy	
			Adjusted: HR (95% CI)	Unadjusted: HR (95% CI)	Adjusted: HR (95% CI)	Unadjusted: HR (95% CI)	Adjusted: HR (95% CI)	Unadjusted: HR (95% CI)
Complete response	1	FEC versus TC	3.54 (2.67, 4.68)	3.57 (2.72, 4.68)	1.30 (0.82, 2.05)	1.15 (0.76, 1.76)	1.02 (0.73, 1.42)	0.99 (0.72, 1.37)
	2	FEC versus TC	2.61 (1.89, 3.60)	3.75 (2.76, 5.10)	0.92 (0.61, 1.40)	1.06 (0.71, 1.56)	1.04 (0.74, 1.46)	1.15 (0.83, 1.59)
	3	FEC versus TC	3.12 (2.26, 4.32)	4.20 (3.08, 5.74)	1.02 (0.63, 1.63)	1.06 (0.68, 1.65)	0.79 (0.56, 1.11)	0.85 (0.61, 1.18)
Total control	1	FEC versus TC	3.31 (2.75, 3.97)	3.25 (2.72, 3.88)	0.95 (0.69, 1.32)	0.91 (0.67, 1.25)	1.21 (0.92, 1.58)	1.17 (0.90, 1.51)
	2	FEC versus TC	3.35 (2.72, 4.14)	4.02 (3.29, 4.93)	1.06 (0.78, 1.43)	1.14 (0.86, 1.53)	0.99 (0.74, 1.32)	1.01 (0.77, 1.34)
	3	FEC versus TC	3.18 (2.58, 3.92)	3.75 (3.07, 4.58)	1.12 (0.78, 1.60)	1.18 (0.83, 1.68)	0.86 (0.64, 1.15)	0.96 (0.72, 1.26)

*Note:* CR HRs were adjusted for predictors alcohol per week, BMI and for the stratification factors age and in cycle 2 and 3 for pre‐cycle event. Smoking was considered in cycle 1 as stratification factor, in cycles 2 and 3 as covariate. Total control HRs were adjusted for predictors alcohol per week, BMI, smoking and pre‐cycle chemo event and for the stratification factor age.

Abbreviations: HR, hazard ratio; CI, confidence interval.

Total control and complete remission were evaluated with regard to different subgroups exploratively. Median nausea‐free times and rates are shown in Tables S1–S4 for age, alcohol consumption, smoking, and BMI subgroups, median emesis‐free times and rates for the same subgroups in Tables S5–S8. The corresponding Kaplan–Meier curves show nausea‐ and emesis‐free survival for those subgroups separately for every cycle [1, 2, 3] in Figures S1–S8 (A–C corresponds to cycles 1–3). Both chemotherapy regimens are displayed separately. Smoking appeared to be the strongest protective factor against nausea‐ and emesis events. Young age, underweight or normal BMI, and alcohol consumption were tendentially risk factors for CINV. Paradoxically, patients under 50 receiving TC had more nausea but fewer emesis events.

## 4 Discussion

In this analysis of patients with early breast cancer, we were able to show that adjuvant chemotherapy with FEC followed by docetaxel leads to increased nausea and emesis compared to TC. This effect was consistently observed over the first three cycles, with the majority of nausea and emesis events occurring within the first 12 h after chemotherapy.

This was reflected by stable HRs in Cox regression analyses. The adjustment for different covariates did not significantly alter the HRs, suggesting that the main factor was the chemotherapy received. With HRs close to 1 in the time frames afterwards (12–24 h, 24–120 h), our study indicates that most initial emetic and nausea events occur early, and that notable differences between the two chemotherapy regimens are identified early on too, in the timeframe 0–12 h after chemotherapy.

The time frame 12–24 h is of special interest, as less events (both emesis and nausea) were recorded. This could be related to patients being asleep, with nausea and emesis less often recorded and possibly also occurring less often during sleep. Nevertheless, a documentation bias may exist, underlining the need for further research. It has previously been reported that poor sleep quality [7] and a late chronotype [5] may increase the probability of CINV. Conversely, nausea and vomiting in pregnancy has been associated with poorer sleep quality [6]. In a vicious circle, poor sleep could then lead to CINV. However, as far as we know, it remains unclear whether CINV may lead to poorer sleep.

The recurrence‐free survival and overall survival rates of the two regimens were described elsewhere [12]. Nowadays, both HRpos/HER2neg breast cancer and triple negative breast cancer are typically treated with neoadjuvant chemotherapy with different therapy regimens, depending on the molecular subtype, demonstrating the rapid development of therapeutic strategies in the field of breast cancer treatment [3].

Although neither FEC nor TC are standard chemotherapy regimens for the treatment of early or advanced breast cancer, both are good therapy options, with TC mentioned in current guidelines when anthracycline‐based chemotherapy is to be avoided [3]. As such, CINV and antiemetic therapy remain a challenge today and will continue to be a challenge with new therapeutic agents such as ADCs. In contrast to conventional chemotherapy, ADCs exhibit prolonged activity, resulting in a prolonged duration of side effects.

Both emesis‐free rates and nausea‐free rates increased with the number of cycles by approximately 10% for each variable in both chemotherapy groups from cycle 1 to cycle 3, possibly indicating suboptimal antiemetic prophylaxes for patients at the start of the chemotherapy. This is consistent with experiences in the daily clinical routine where managing side effects is sometimes not the primary focus of attending physicians. With TC alone, the emesis‐free rate increased by only 2% (from 73% in cycle 1 to 75% in cycle 3), which may indicate a ceiling when antiemetic therapy cannot be improved further. Interestingly, for FEC, 74% of all nausea and 68% of all emetic events occurred during the first 12 h (cycle 1), while this number is much lower for TC (60% of nausea and 33% of emetic events).

The reported mean terminal elimination half‐lives for the given regimens are approximately 8 h for 5‐fluorouracil [16], 25–33 h for epirubicin [17], 6–12 h for cyclophosphamide [18], and about 12 h for docetaxel [19]. Thus, pharmacokinetics cannot primarily explain the observed differences from 12 h post‐chemotherapy onwards. According to the protocol, an NK1 receptor antagonist was recommended for patients receiving FEC. Aprepitant, which was predominantly used, is administered during the first 3 days of the chemotherapy cycle. This may explain the ceiling effect of CINV in patients receiving FEC, as the chemotherapeutic agents are largely cleared from the body over time, while the antiemetic medication remains active for an extended period. For patients receiving TC, only dexamethasone was recommended once on the first day after chemotherapy, which may indicate insufficient antiemetic coverage during the initial days following infusion.

Although other NK1 receptor antagonists were theoretically available, only aprepitant and fosaprepitant were accessible during the study period. Aprepitant was documented in all but one case in the raw data as the administered NK1 receptor antagonist (fosaprepitant was used once). Documentation of specific antiemetic agents was poor, despite clear recommendations for antiemetic prophylaxis according to the study protocol. As persistence rates below 30% for antiemetic agents appear to be unrealistic, a documentation bias regarding antiemetic drugs seems probable.

Based on the recommended antiemetic therapy and the observed nausea and vomiting events in the days following chemotherapy administration, it could be assumed that antiemetic therapy was insufficient in the TC group. A clinical takeaway from this evaluation would be the possibility of offering extended antiemetic therapy to this patient group in case of clinical presentations of nausea on and beyond day one after chemotherapy infusion.

Compared to literature, the overall emesis‐free rate (120 h) in our study was lower for TC. Llobart et al. reported an overall CR of 87% (95% CI, 82–92) in a prospective study with 211 patients [20], compared to 73% (95% CI, 0.69, 0.76) reported in our study in cycle 1. For FEC, Bonneterre et al. reported control of emesis in 35 patients receiving FEC or FAC (anthracycline or epirubicin plus 5‐fluorouracil and cyclophosphamide) and ondansetron as prophylactic antiemetic therapy in 66% of patients in the first 24 h, compared to 70% (95% CI, 0.67, 0.73) [21]. While the numbers are comparable, this study is an older one from 1990, with only a small number of patients. In another study with 114 patients, acute nausea was reported in 58% of patients, which is comparable to the reported 67% (95% CI, 0.63, 0.70) in our cohort. Nevertheless, that study also had a limited patient number and daily nausea evaluation was conducted with telephone calls with the retrospective evaluation of nausea from the previous day on a daily resolution basis, leaving room for recall bias [22]. Therefore, CINV may be under‐reported in older studies and is a real problem that negatively affects the quality of life of patients undergoing chemotherapy.

With regard to the subgroups, the observed effects were mostly in line with previously published publications, thus confirming known risk factors for CINV [23]. Young patient age was linked to higher rates of nausea, while emesis was not influenced by the patient's age in the TC group. Young patients' age being a risk factor for chemotherapy induced nausea was reported before [24]. Chemotherapy‐induced emesis associated with younger patient age has been discussed previously. However, most studies report risk factors primarily in relation to chemotherapy‐induced nausea, leaving the emesis component underreported [24, 25, 26]. With FEC, younger patients indeed experience increased emesis; this is not observed with patients receiving TC. The pathophysiological mechanisms leave room for speculation, highlighting that risk factors for CINV are not yet fully understood and that further research is necessary.

A limitation of this analysis is that CINV was not the focus of the SUCCESS C study, which primarily evaluated the efficacy of the two chemotherapy regimens and, secondarily, included a randomized lifestyle intervention after completion of the chemotherapy. Furthermore, although prophylactic antiemetic therapy was predefined per protocol, its administration—particularly in the days following chemotherapy—could no longer be monitored by the study personnel. An additional concern is potential underreporting in the patient diaries regarding the administration of prophylactic antiemetic treatment. As patients were not blinded for the regimen received, a potential expectancy towards more or less CINV is not discardable and has to be mentioned as a potential bias.

The main strength of the study is the large number of patients and the high temporal resolution, with hourly assessments of nausea and vomiting. The comparison of two different chemotherapy regimens allows a profound statement about the emetogenic effect of the regimens used. As described in the literature and analogue to recommendations that classify FEC as highly emetogenic and TC as moderately emetogenic, our analysis shows a consistent and statistically significant difference between the two chemotherapy regimens regarding CINV. Prophylactic antiemetic therapy could not eliminate the higher occurrence of emetic events in patients receiving FEC compared to TC.

Few reliable emesis‐free rates or nausea‐free rates were found in literature for the chemotherapy regimens used, highlighting the need for further research into CINV and the value of this study.

## 5 Conclusion

In conclusion, a difference in CINV was found between the two chemotherapy regimens FEC followed by docetaxel versus TC, with higher overall emesis‐free and nausea‐free rates for patients receiving TC. This observation was consistent over all three evaluated cycles with better nausea and emesis control in cycles 2 and 3. The time frame 0–12 h showed a significant difference between the two regimens in both CR and total control. Between 12–24 h and 24–120 h, no difference regarding CR and total control was observed, possibly linked to the short half‐lives of the chemotherapeutic agents.

This is the first study to evaluate CINV for a big patient collective with high temporal resolution using modern antiemetic treatments and high‐quality data for patients receiving FEC or TC. Our results demonstrate that chemotherapy regimens have varying emetogenic patterns, with critical differences appearing within the first 12 h after treatment, questioning the conventional classification of acute and delayed CINV. Further CINV research is needed, focusing on a high temporal resolution of CINV evaluation and associated co‐factors such as sleep, depression, and eating and drinking, particularly considering the new ADCs, which have brought CINV back into focus as a relevant side effect.

## Author Contributions

**Manuel Hörner:** conceptualization, writing – original draft, writing – review and editing, methodology, investigation, visualization. **Henning Schäffler:** writing – review and editing, investigation. **Lothar Häberle:** writing – review and editing, investigation, supervision, methodology, validation. **Chloë Goossens:** writing – review and editing, investigation. **Kerstin Pfister:** writing – review and editing, investigation. **Elena Leinert:** writing – review and editing, investigation. **Kristina Veselinovic:** writing – review and editing, investigation. **Sara Y. Brucker:** writing – review and editing, investigation. **Uwe Köhler:** writing – review and editing, investigation. **Georg Heinrich:** writing – review and editing, investigation. **Andreas Schneeweiss:** writing – review and editing, investigation. **Matthias W. Beckmann:** writing – review and editing, investigation. **Peter A. Fasching:** writing – review and editing, conceptualization, methodology, project administration, resources, supervision, validation. **Wolfgang Janni:** writing – review and editing, project administration, investigation, resources. **Brigitte Rack:** writing – review and editing, investigation. **Sabine Heublein:** writing – review and editing, investigation. **Philipp Ziegler:** writing – review and editing, conceptualization, methodology, investigation, data curation, visualization, writing – original draft, formal analysis, software.

## Funding

The SUCCESS C trial was an investigator‐sponsored trial. The overhead of the study was partly supported by the pharmaceutical companies Sanofi‐Aventis, Pfizer, Chugai, and GlaxoSmithKline. This analysis received no external funding.

## Ethics Statement

The study was approved by the Heinrich Heine University Düsseldorf Ethics Committee (ethics approval number: MC‐LKP‐319) and registered under the EU Clinical Trials Register https://www.clinicaltrialsregister.eu/, identifier: 2008‐005453‐38. Written consent was obtained from all participants.

## Conflicts of Interest

M.H. received research support from Helsinn Healthcare SA, honoraria from Lilly Deutschland GmbH, Thieme and travel support from Novartis, Lilly Deutschland GmbH, and AstraZeneca; H.S. received honoraria from Pfizer, Novartis, GILEAD, Lilly, Daiichi, Eickeler Veranstaltungen, Novartis, GILEAD; C.G. received speaker honoraria from Novartis Pharma GmbH and ClinSol GmbH & Co. KG.; K.P. received honoraria from Pfizer, Novartis, Gilead and travel support from Novartis; S.Y.B. received advisory board honoraria from Lilly und Astra Zeneca; A.S. received honoraria from Amgen, AstraZeneca, Aurikamed, Bayer, Celgene, ClinSol, Clovis Oncology, coma, UroGyn, Connectmedica, Daiichi Sankyo, Gilead, GSK, if‐kongress, I‐MED, iOMEDICO, Lilly, MCI Deutschland, med publico, Menarini Stemline, Metaplan, MSD, Mylan, NanoString Technologies, Novartis, onkowissen.de, Pfizer, Pierre Fabre, promedicis, Roche, Seagen, streamedup, SYNLAB, Tesaro and travel support from AstraZeneca, Celgene, Daiichi Sankyo, Gilead, Pfizer, Roche; P.A.F. has received institutional funding from BioNTech, Cepheid and Pfizer and personal honoraria from Novartis, Pfizer, Roche, Daiichi Sankyo, AstraZeneca, Lilly, Eisai, Merck Sharp & Dohme, Pierre Fabre, SeaGen, Agendia, Sanofi Aventis, Gilead and Mylan for consulting, participation in advisory boards and steering committees and/or lectures. His institution conducts research for Novartis; W.J. received research Grants and/or honoraria from AstraZeneca, Cellgene, Chugai, DaiichiSankyo, Eisai, ExactScience, Gilead, GSK, Guardant Health, Janssen, Lilly, Menarini Stemline, MSD, NeoGenomics, Novartis, Pfizer, Roche, Sanofi‐Aventis, Seagen; B.R. received institutional research funding from Sanofi‐Aventis, Novartis, Lilly and Chugai and honoraria from Lilly; S.H. received honoraria from MSD, Novartis, Roche, AstraZeneca, Pfizer, Gilead, Daichi Sankyo, AbbVie, GSK, Clovis, Immunogen, Pharmavid and for participation in advisory boards from MSD, Novartis, Roche, AstraZeneca, Daichi Sankyo, AbbVie, GSK, Immunogen, Pfizer; The other authors declare no conflicts of interest.

## Supporting information


**Table S1:** Median nausea‐event‐free survival times and survival rates relative to cycle and Age subgroup.
**Table S2:** Median nausea‐event‐free survival times and survival rates relative to cycle and smoking subgroup.
**Table S3:** Median nausea‐event‐free survival times and survival rates relative to cycle and drinking subgroup.
**Table S4:** Median nausea‐event‐free survival times and survival rates relative to cycle and BMI subgroup.
**Table S5:** Emetic‐event‐free survival rates relative to cycle and Age subgroup.
**Table S6:** Emetic‐event‐free survival rates relative to cycle and smoking subgroup.
**Table S7:** Emetic‐event‐free survival rates relative to cycle and drinking subgroup.
**Table S8:** Emetic‐event‐free survival rates relative to cycle and BMI subgroup.
**Figure S1:** Nausea‐free survival described via Kaplan Meier curves between the FEC and the TC group for age subgroups.
**Figure S2:** Nausea‐free survival described via Kaplan Meier curves between the FEC and the TC group for smoking subgroups.
**Figure S3:** Nausea‐free survival described via Kaplan Meier curves between the FEC and the TC group for alcohol subgroups.
**Figure S4:** Nausea‐free survival described via Kaplan Meier curves between the FEC and the TC group for BMI subgroups. A shows cycle 1, B cycle 2, and C cycle 3.
**Figure S5:** Emesis‐free survival described via Kaplan Meier curves between the FEC and the TC group for age subgroups. A shows cycle 1, B cycle 2, and C cycle 3.
**Figure S6:** Emesis‐free survival described via Kaplan Meier curves between the FEC and the TC group for smoking subgroups. A shows cycle 1, B cycle 2, and C cycle 3.
**Figure S7:** Emesis‐free survival described via Kaplan Meier curves between the FEC and the TC group for alcohol subgroups. A shows cycle 1, B cycle 2, and C cycle 3.
**Figure S8:** Emesis‐free survival described via Kaplan Meier curves between the FEC and the TC group for BMI subgroups. A shows cycle 1, B cycle 2, and C cycle 3.

## Data Availability

The datasets used and/or analyzed in this study are available to researchers with proposals regarding chemotherapy‐induced nausea and vomiting, after approval from the SUCCESS C scientific board. Further information is available from the corresponding author upon request.
