# Supplementary material for: Chemotherapy‐Induced Nausea and Vomiting in Early Breast Cancer Patients Receiving Adjuvant Chemotherapy With Fluorouracil, Epirubicin, Cyclophosphamide Followed by Docetaxel Versus an Anthracycline‐Free Regimen With Docetaxel, Cyclophosphamide—Results From a Randomized Clinical Trial
Source: Int J Cancer. 2026 Apr 29;159(8):1990–2003. doi: 10.1002/ijc.70528 (PMC13495824; doi:10.1002/ijc.70528)
Supplement: Supplementary file 1 [file IJC-159-1990-s001.pdf]

Supplementary Material

Chemotherapy-induced nausea and vomiting in early breast cancer patients receiving adjuvant chemotherapy with FEC followed by docetaxel versus an anthracycline-free regimen with docetaxel/cyclophosphamide – results from a randomized clinical trial

Manuel Hörner, Henning Schäffler, Lothar Häberle, Chloë Goossens, Kerstin Pfister, Elena Leinert, Kristina Veselinovic, Sara Brucker, Uwe Köhler, Georg Heinrich, Andreas Schneeweiss, Matthias W. Beckmann, Peter A. Fasching, Wolfgang W. Janni, Brigitte Rack, Sabine Heublein, Philipp Ziegler

Table of contents

Supplemental table 1. Median nausea-event-free survival times and survival rates relative to cycle and Age subgroup. 2

Supplemental table 2. Median nausea-event-free survival times and survival rates relative to cycle and smoking subgroup. 3

Supplemental table 3. Median nausea-event-free survival times and survival rates relative to cycle and drinking subgroup. 4

Supplemental table 4. Median nausea-event-free survival times and survival rates relative to cycle and BMI subgroup. 5

Supplemental table 5. Emetic-event-free survival rates relative to cycle and Age subgroup. 6

Supplemental table 6. Emetic-event-free survival rates relative to cycle and smoking subgroup. 7

Supplemental table 7. Emetic-event-free survival rates relative to cycle and drinking subgroup. 8

Supplemental table 8. Emetic-event-free survival rates relative to cycle and BMI subgroup. 9

Supplemental figure 1. Nausea-free survival described via Kaplan Meier curves between the FEC and the TC group for age subgroups. 10

Supplemental figure 2. Nausea-free survival described via Kaplan Meier curves between the FEC and the TC group for smoking subgroups. 13

Supplemental figure 3. Nausea-free survival described via Kaplan Meier curves between the FEC and the TC group for alcohol subgroups. 16

Supplemental figure 4. Nausea-free survival described via Kaplan Meier curves between the FEC and the TC group for BMI subgroups. A shows cycle 1, B cycle 2, and C cycle 3. 19

Supplemental figure 5. Emesis-free survival described via Kaplan Meier curves between the FEC and the TC group for age subgroups. A shows cycle 1, B cycle 2, and C cycle 3. 22

Supplemental figure 6. Emesis-free survival described via Kaplan Meier curves between the FEC and the TC group for smoking subgroups. A shows cycle 1, B cycle 2, and C cycle 3. 25

Supplemental figure 7. Emesis-free survival described via Kaplan Meier curves between the FEC and the TC group for alcohol subgroups. A shows cycle 1, B cycle 2, and C cycle 3. 28

Supplemental figure 8. Emesis-free survival described via Kaplan Meier curves between the FEC and the TC group for BMI subgroups. A shows cycle 1, B cycle 2, and C cycle 3. 31

Supplemental table 1. Median nausea-event-free survival times and survival rates relative to cycle and Age subgroup.

Cycle	Age	Therapy	Patients	Events	Median nausea-free time in hours (95% CI)	12-hour nausea-free rate (95% CI)	24-hour nausea-free rate (95% CI)	48-hour nausea-free rate (95% CI)	120-hour nausea-free rate (95% CI)
1	age<50	TC	240	141	54.5 (27.0, 100.0)	0.74 (0.68, 0.80)	0.57 (0.51, 0.64)	0.51 (0.45, 0.58)	0.41 (0.35, 0.48)
1	age<50	FEC	243	204	7.0 (7.0, 8.0)	0.27 (0.22, 0.33)	0.23 (0.19, 0.29)	0.20 (0.15, 0.25)	0.16 (0.12, 0.21)
1	age 50-64	TC	344	207	55.0 (43.0, 82.0)	0.75 (0.71, 0.80)	0.60 (0.55, 0.66)	0.53 (0.48, 0.58)	0.40 (0.35, 0.45)
1	age 50-64	FEC	383	299	10.0 (9.0, 11.0)	0.44 (0.39, 0.49)	0.35 (0.31, 0.41)	0.28 (0.24, 0.33)	0.22 (0.18, 0.26)
1	age 65+	TC	147	88	90.0 (44.0, 119.0)	0.84 (0.79, 0.90)	0.67 (0.59, 0.75)	0.55 (0.48, 0.64)	0.40 (0.33, 0.49)
1	age 65+	FEC	134	96	21.0 (13.0, 32.0)	0.58 (0.50, 0.67)	0.44 (0.36, 0.53)	0.37 (0.30, 0.46)	0.28 (0.21, 0.37)
2	age<50	TC	239	130	72.0 (48.0, NA)	0.81 (0.76, 0.86)	0.64 (0.58, 0.70)	0.55 (0.49, 0.62)	0.46 (0.40, 0.52)
2	age<50	FEC	244	195	8.0 (8.0, 9.0)	0.34 (0.28, 0.40)	0.26 (0.21, 0.32)	0.22 (0.17, 0.28)	0.20 (0.15, 0.25)
2	age 50-64	TC	342	173	94.0 (69.0, NA)	0.82 (0.78, 0.86)	0.67 (0.63, 0.73)	0.61 (0.56, 0.67)	0.49 (0.44, 0.55)
2	age 50-64	FEC	382	272	12.0 (10.0, 19.0)	0.49 (0.45, 0.55)	0.39 (0.35, 0.44)	0.33 (0.29, 0.38)	0.29 (0.24, 0.34)
2	age 65+	TC	144	74	114.0 (69.0, NA)	0.88 (0.83, 0.94)	0.71 (0.64, 0.79)	0.65 (0.58, 0.74)	0.48 (0.41, 0.57)
2	age 65+	FEC	133	94	22.0 (15.0, 46.0)	0.59 (0.52, 0.68)	0.46 (0.38, 0.55)	0.39 (0.32, 0.49)	0.28 (0.22, 0.37)
3	age<50	TC	241	121	105.0 (54.0, NA)	0.81 (0.76, 0.86)	0.69 (0.63, 0.75)	0.57 (0.51, 0.64)	0.49 (0.43, 0.56)
3	age<50	FEC	239	178	8.0 (7.0, 9.0)	0.35 (0.29, 0.41)	0.29 (0.24, 0.36)	0.27 (0.22, 0.33)	0.25 (0.20, 0.31)
3	age 50-64	TC	340	152	NA (116.0, NA)	0.81 (0.77, 0.86)	0.73 (0.68, 0.77)	0.64 (0.59, 0.69)	0.55 (0.50, 0.60)
3	age 50-64	FEC	378	267	11.0 (10.0, 21.0)	0.49 (0.44, 0.54)	0.42 (0.38, 0.48)	0.38 (0.33, 0.43)	0.29 (0.25, 0.34)
3	age 65+	TC	143	69	NA (79.0, NA)	0.87 (0.81, 0.92)	0.75 (0.68, 0.82)	0.68 (0.61, 0.76)	0.51 (0.44, 0.60)
3	age 65+	FEC	129	78	47.0 (22.0, 111.0)	0.66 (0.58, 0.75)	0.55 (0.47, 0.64)	0.49 (0.41, 0.59)	0.39 (0.31, 0.49)

Supplemental table 2. Median nausea-event-free survival times and survival rates relative to cycle and smoking subgroup.

Cycle	Smoking	Therapy	Patients	Events	Median nausea-free time in hours (95% CI)	12-hour nausea-free rate (95% CI)	24-hour nausea-free rate (95% CI)	48-hour nausea-free rate (95% CI)	120-hour nausea-free rate (95% CI)
1	smoker	TC	64	33	99.0 (36.0, NA)	0.81 (0.72, 0.91)	0.67 (0.56, 0.80)	0.59 (0.48, 0.72)	0.48 (0.37, 0.62)
1	smoker	FEC	74	46	31.0 (18.0, NA)	0.62 (0.52, 0.74)	0.53 (0.42, 0.65)	0.47 (0.37, 0.60)	0.38 (0.28, 0.51)
1	non-smoker	TC	616	368	66.0 (47.0, 83.0)	0.77 (0.74, 0.80)	0.60 (0.57, 0.64)	0.53 (0.49, 0.57)	0.40 (0.36, 0.44)
1	non-smoker	FEC	617	495	9.0 (8.0, 9.0)	0.38 (0.35, 0.42)	0.31 (0.27, 0.35)	0.24 (0.21, 0.28)	0.19 (0.16, 0.23)
2	smoker	TC	65	31	NA (59.0, NA)	0.86 (0.78, 0.95)	0.71 (0.60, 0.83)	0.66 (0.55, 0.78)	0.52 (0.41, 0.66)
2	smoker	FEC	74	36	NA (31.0, NA)	0.70 (0.61, 0.81)	0.63 (0.53, 0.75)	0.56 (0.46, 0.69)	0.51 (0.41, 0.64)
2	non-smoker	TC	613	315	94.0 (72.0, NA)	0.83 (0.80, 0.86)	0.67 (0.63, 0.71)	0.60 (0.56, 0.64)	0.48 (0.45, 0.53)
2	non-smoker	FEC	616	473	9.5 (9.0, 11.0)	0.44 (0.40, 0.48)	0.33 (0.30, 0.37)	0.28 (0.24, 0.32)	0.23 (0.20, 0.26)
3	smoker	TC	65	29	NA (43.0, NA)	0.85 (0.76, 0.94)	0.72 (0.62, 0.84)	0.57 (0.46, 0.71)	0.54 (0.43, 0.68)
3	smoker	FEC	73	38	102.0 (48.0, NA)	0.67 (0.57, 0.79)	0.62 (0.51, 0.74)	0.59 (0.49, 0.71)	0.48 (0.38, 0.61)
3	non-smoker	TC	612	289	NA (98.0, NA)	0.82 (0.79, 0.85)	0.72 (0.68, 0.76)	0.63 (0.60, 0.67)	0.53 (0.49, 0.57)
3	non-smoker	FEC	608	439	9.0 (8.0, 11.0)	0.45 (0.41, 0.49)	0.38 (0.34, 0.42)	0.33 (0.30, 0.37)	0.27 (0.24, 0.31)

Supplemental table 3. Median nausea-event-free survival times and survival rates relative to cycle and drinking subgroup.

Cycle	Drinking	Therapy	Patients	Events	Median nausea-free time in hours (95% CI)	12-hour nausea-free rate (95% CI)	24-hour nausea-free rate (95% CI)	48-hour nausea-free rate (95% CI)	120-hour nausea-free rate (95% CI)
1	never drinking	TC	294	163	82.0 (60.0, NA)	0.77 (0.72, 0.82)	0.63 (0.58, 0.69)	0.57 (0.52, 0.63)	0.45 (0.39, 0.51)
1	never drinking	FEC	320	258	9.0 (8.0, 10.0)	0.39 (0.34, 0.45)	0.31 (0.26, 0.36)	0.26 (0.21, 0.31)	0.19 (0.15, 0.24)
1	drinking 1-2 days/week	TC	295	189	45.0 (29.0, 72.0)	0.75 (0.70, 0.80)	0.58 (0.53, 0.64)	0.48 (0.43, 0.54)	0.36 (0.31, 0.42)
1	drinking 1-2 days/week	FEC	320	247	9.0 (8.0, 10.0)	0.41 (0.36, 0.47)	0.35 (0.30, 0.41)	0.28 (0.24, 0.33)	0.23 (0.18, 0.28)
1	drinking 3-7 days/week	TC	85	50	67.0 (27.0, NA)	0.78 (0.69, 0.87)	0.60 (0.50, 0.71)	0.54 (0.44, 0.66)	0.41 (0.32, 0.53)
1	drinking 3-7 days/week	FEC	75	61	9.0 (7.0, 18.0)	0.44 (0.34, 0.57)	0.33 (0.24, 0.46)	0.23 (0.15, 0.34)	0.19 (0.12, 0.30)
2	never drinking	TC	294	150	95.0 (72.0, NA)	0.84 (0.80, 0.88)	0.67 (0.62, 0.73)	0.63 (0.58, 0.69)	0.49 (0.44, 0.55)
2	never drinking	FEC	317	234	11.0 (9.0, 16.0)	0.46 (0.41, 0.52)	0.35 (0.30, 0.40)	0.30 (0.25, 0.36)	0.25 (0.21, 0.31)
2	drinking 1-2 days/week	TC	291	156	70.0 (52.0, NA)	0.81 (0.77, 0.86)	0.65 (0.60, 0.71)	0.57 (0.51, 0.63)	0.46 (0.40, 0.52)
2	drinking 1-2 days/week	FEC	322	244	10.0 (8.0, 15.0)	0.45 (0.40, 0.51)	0.36 (0.31, 0.42)	0.29 (0.24, 0.34)	0.24 (0.20, 0.29)
2	drinking 3-7 days/week	TC	85	44	95.0 (47.0, NA)	0.81 (0.73, 0.90)	0.66 (0.57, 0.77)	0.59 (0.49, 0.70)	0.48 (0.38, 0.60)
2	drinking 3-7 days/week	FEC	76	56	10.0 (8.0, 36.0)	0.47 (0.37, 0.60)	0.38 (0.29, 0.51)	0.36 (0.26, 0.48)	0.26 (0.18, 0.38)
3	never drinking	TC	292	137	NA (92.0, NA)	0.84 (0.80, 0.88)	0.71 (0.66, 0.76)	0.63 (0.58, 0.69)	0.53 (0.47, 0.59)
3	never drinking	FEC	310	217	11.0 (9.0, 20.0)	0.48 (0.43, 0.54)	0.40 (0.35, 0.46)	0.36 (0.31, 0.42)	0.30 (0.25, 0.35)
3	drinking 1-2 days/week	TC	290	140	NA (71.0, NA)	0.82 (0.78, 0.87)	0.73 (0.68, 0.78)	0.60 (0.55, 0.66)	0.51 (0.46, 0.57)
3	drinking 1-2 days/week	FEC	318	227	10.0 (8.0, 18.0)	0.46 (0.41, 0.52)	0.39 (0.34, 0.45)	0.34 (0.29, 0.40)	0.28 (0.24, 0.34)
3	drinking 3-7 days/week	TC	85	34	NA (NA, NA)	0.75 (0.67, 0.85)	0.68 (0.59, 0.79)	0.65 (0.55, 0.76)	0.60 (0.50, 0.71)
3	drinking 3-7 days/week	FEC	75	52	17.0 (8.0, 53.0)	0.52 (0.42, 0.65)	0.44 (0.34, 0.57)	0.40 (0.30, 0.53)	0.30 (0.21, 0.43)

Supplemental table 4. Median nausea-event-free survival times and survival rates relative to cycle and BMI subgroup.

Cycle	BMI	Therapy	Patients	Events	Median nausea-free time in hours (95% CI)	12-hour nausea-free rate (95% CI)	24-hour nausea-free rate (95% CI)	48-hour nausea-free rate (95% CI)	120-hour nausea-free rate (95% CI)
1	underweight/normal	TC	333	199	54.0 (36.0, 80.0)	0.76 (0.72, 0.81)	0.59 (0.53, 0.64)	0.51 (0.46, 0.56)	0.40 (0.35, 0.46)
1	underweight/normal	FEC	354	284	8.0 (8.0, 10.0)	0.38 (0.34, 0.44)	0.32 (0.27, 0.37)	0.25 (0.21, 0.30)	0.19 (0.16, 0.24)
1	overweight	TC	244	141	78.0 (45.0, 115.0)	0.77 (0.72, 0.83)	0.62 (0.56, 0.69)	0.55 (0.49, 0.61)	0.42 (0.36, 0.49)
1	overweight	FEC	253	196	10.0 (8.0, 13.0)	0.44 (0.39, 0.51)	0.34 (0.28, 0.40)	0.28 (0.23, 0.34)	0.22 (0.18, 0.28)
1	obese	TC	154	96	56.0 (42.0, 100.0)	0.75 (0.69, 0.82)	0.62 (0.54, 0.70)	0.54 (0.47, 0.62)	0.37 (0.30, 0.46)
1	obese	FEC	153	119	9.0 (9.0, 12.0)	0.41 (0.34, 0.50)	0.36 (0.29, 0.44)	0.30 (0.23, 0.38)	0.22 (0.16, 0.30)
2	underweight/normal	TC	331	181	72.0 (50.0, NA)	0.81 (0.77, 0.85)	0.62 (0.57, 0.68)	0.55 (0.50, 0.61)	0.45 (0.40, 0.51)
2	underweight/normal	FEC	354	265	9.0 (8.0, 11.0)	0.43 (0.38, 0.48)	0.32 (0.28, 0.38)	0.28 (0.23, 0.33)	0.25 (0.20, 0.30)
2	overweight	TC	242	121	119.0 (72.0, NA)	0.86 (0.81, 0.90)	0.71 (0.66, 0.77)	0.65 (0.59, 0.71)	0.50 (0.44, 0.56)
2	overweight	FEC	252	187	12.0 (9.0, 19.0)	0.50 (0.44, 0.56)	0.39 (0.33, 0.45)	0.32 (0.27, 0.39)	0.26 (0.21, 0.32)
2	obese	TC	152	75	NA (68.0, NA)	0.84 (0.78, 0.90)	0.70 (0.63, 0.78)	0.63 (0.56, 0.71)	0.50 (0.43, 0.59)
2	obese	FEC	153	109	11.0 (9.0, 23.0)	0.48 (0.41, 0.57)	0.40 (0.33, 0.48)	0.34 (0.28, 0.43)	0.28 (0.22, 0.37)
3	underweight/normal	TC	330	167	85.0 (59.0, NA)	0.79 (0.75, 0.84)	0.67 (0.62, 0.72)	0.56 (0.51, 0.62)	0.49 (0.44, 0.55)
3	underweight/normal	FEC	349	253	9.0 (8.0, 11.0)	0.43 (0.38, 0.48)	0.36 (0.31, 0.41)	0.32 (0.28, 0.38)	0.27 (0.23, 0.32)
3	overweight	TC	242	109	NA (98.0, NA)	0.85 (0.80, 0.89)	0.76 (0.70, 0.81)	0.66 (0.61, 0.73)	0.55 (0.49, 0.61)
3	overweight	FEC	249	178	11.0 (9.0, 23.0)	0.49 (0.44, 0.56)	0.43 (0.37, 0.50)	0.38 (0.33, 0.45)	0.28 (0.23, 0.34)
3	obese	TC	152	66	NA (96.0, NA)	0.85 (0.79, 0.91)	0.76 (0.69, 0.83)	0.69 (0.62, 0.77)	0.56 (0.48, 0.64)
3	obese	FEC	148	92	21.0 (10.0, 54.0)	0.55 (0.47, 0.63)	0.46 (0.39, 0.55)	0.42 (0.34, 0.50)	0.37 (0.30, 0.46)

Supplemental table 5. Emetic-event-free survival rates relative to cycle and Age subgroup.

Cycle	Age	Therapy	Patients	Events	12-hour emesis-free rate (95% CI)	24-hour emesis-free rate (95% CI)	48-hour emesis-free rate (95% CI)	120-hour emesis-free rate (95% CI)
1	age<50	TC	240	53	0.93 (0.89, 0.96)	0.88 (0.83, 0.92)	0.86 (0.82, 0.91)	0.78 (0.73, 0.83)
1	age<50	FEC	243	121	0.60 (0.54, 0.66)	0.56 (0.50, 0.63)	0.54 (0.48, 0.60)	0.50 (0.44, 0.57)
1	age 50-64	TC	344	102	0.88 (0.85, 0.92)	0.82 (0.78, 0.86)	0.80 (0.75, 0.84)	0.70 (0.66, 0.75)
1	age 50-64	FEC	383	163	0.73 (0.69, 0.78)	0.68 (0.63, 0.73)	0.63 (0.58, 0.68)	0.57 (0.53, 0.62)
1	age 65+	TC	147	45	0.93 (0.89, 0.97)	0.85 (0.79, 0.91)	0.81 (0.75, 0.88)	0.69 (0.62, 0.77)
1	age 65+	FEC	134	52	0.80 (0.73, 0.87)	0.70 (0.63, 0.78)	0.67 (0.60, 0.76)	0.61 (0.53, 0.70)
2	age<50	TC	239	50	0.94 (0.91, 0.97)	0.87 (0.83, 0.91)	0.84 (0.79, 0.88)	0.79 (0.74, 0.84)
2	age<50	FEC	244	114	0.64 (0.59, 0.71)	0.60 (0.54, 0.67)	0.57 (0.51, 0.64)	0.53 (0.47, 0.60)
2	age 50-64	TC	342	90	0.92 (0.89, 0.95)	0.85 (0.82, 0.89)	0.82 (0.78, 0.87)	0.73 (0.69, 0.78)
2	age 50-64	FEC	382	136	0.79 (0.75, 0.83)	0.73 (0.68, 0.77)	0.69 (0.65, 0.74)	0.64 (0.60, 0.69)
2	age 65+	TC	144	40	0.94 (0.91, 0.98)	0.83 (0.77, 0.90)	0.81 (0.75, 0.88)	0.72 (0.65, 0.80)
2	age 65+	FEC	133	55	0.84 (0.78, 0.91)	0.75 (0.68, 0.83)	0.68 (0.61, 0.77)	0.58 (0.50, 0.67)
3	age<50	TC	241	54	0.95 (0.92, 0.98)	0.89 (0.85, 0.93)	0.85 (0.80, 0.89)	0.77 (0.72, 0.83)
3	age<50	FEC	239	105	0.66 (0.60, 0.72)	0.62 (0.56, 0.69)	0.60 (0.54, 0.67)	0.56 (0.50, 0.63)
3	age 50-64	TC	340	84	0.92 (0.89, 0.95)	0.86 (0.82, 0.90)	0.83 (0.79, 0.87)	0.75 (0.71, 0.80)
3	age 50-64	FEC	378	139	0.76 (0.72, 0.81)	0.71 (0.67, 0.76)	0.68 (0.63, 0.73)	0.63 (0.58, 0.68)
3	age 65+	TC	143	40	0.94 (0.90, 0.98)	0.87 (0.82, 0.93)	0.82 (0.76, 0.89)	0.72 (0.65, 0.80)
3	age 65+	FEC	129	47	0.81 (0.75, 0.88)	0.75 (0.68, 0.83)	0.72 (0.65, 0.80)	0.63 (0.55, 0.72)

Supplemental table 6. Emetic-event-free survival rates relative to cycle and smoking subgroup.

Cycle	Smoking	Therapy	Patients	Events	12-hour emesis-free rate (95% CI)	24-hour emesis-free rate (95% CI)	48-hour emesis-free rate (95% CI)	120-hour emesis-free rate (95% CI)
1	smoker	TC	64	9	0.94 (0.88, 1.00)	0.91 (0.84, 0.98)	0.91 (0.84, 0.98)	0.86 (0.78, 0.95)
1	smoker	FEC	74	28	0.82 (0.74, 0.92)	0.77 (0.68, 0.87)	0.70 (0.61, 0.81)	0.62 (0.52, 0.74)
1	non-smoker	TC	616	170	0.91 (0.89, 0.94)	0.85 (0.82, 0.88)	0.82 (0.79, 0.85)	0.72 (0.69, 0.76)
1	non-smoker	FEC	617	280	0.68 (0.65, 0.72)	0.63 (0.59, 0.66)	0.59 (0.55, 0.63)	0.54 (0.51, 0.59)
2	smoker	TC	65	10	0.94 (0.88, 1.00)	0.91 (0.84, 0.98)	0.88 (0.80, 0.96)	0.84 (0.76, 0.94)
2	smoker	FEC	74	17	0.91 (0.84, 0.97)	0.82 (0.74, 0.92)	0.80 (0.71, 0.89)	0.77 (0.68, 0.87)
2	non-smoker	TC	613	154	0.93 (0.91, 0.95)	0.85 (0.82, 0.88)	0.83 (0.80, 0.86)	0.75 (0.71, 0.78)
2	non-smoker	FEC	616	259	0.74 (0.71, 0.78)	0.68 (0.64, 0.71)	0.63 (0.60, 0.67)	0.58 (0.54, 0.62)
3	smoker	TC	65	12	0.95 (0.90, 1.00)	0.91 (0.84, 0.98)	0.87 (0.80, 0.96)	0.81 (0.72, 0.91)
3	smoker	FEC	73	19	0.88 (0.80, 0.96)	0.84 (0.75, 0.93)	0.79 (0.71, 0.89)	0.74 (0.65, 0.85)
3	non-smoker	TC	612	151	0.93 (0.91, 0.95)	0.87 (0.84, 0.90)	0.83 (0.80, 0.86)	0.75 (0.72, 0.79)
3	non-smoker	FEC	608	243	0.72 (0.69, 0.76)	0.68 (0.64, 0.71)	0.65 (0.61, 0.68)	0.60 (0.56, 0.64)

Supplemental table 7. Emetic-event-free survival rates relative to cycle and drinking subgroup.

Cycle	Drinking	Therapy	Patients	Events	12-hour emesis-free rate (95% CI)	24-hour emesis-free rate (95% CI)	48-hour emesis-free rate (95% CI)	120-hour emesis-free rate (95% CI)
1	never drinking	TC	294	80	0.89 (0.85, 0.92)	0.83 (0.79, 0.88)	0.81 (0.77, 0.86)	0.73 (0.68, 0.78)
1	never drinking	FEC	320	157	0.67 (0.62, 0.72)	0.61 (0.56, 0.67)	0.58 (0.52, 0.63)	0.51 (0.45, 0.56)
1	drinking 1-2 days/week	TC	295	80	0.91 (0.88, 0.94)	0.85 (0.81, 0.89)	0.82 (0.78, 0.87)	0.73 (0.68, 0.78)
1	drinking 1-2 days/week	FEC	320	125	0.73 (0.68, 0.78)	0.68 (0.63, 0.73)	0.64 (0.59, 0.69)	0.61 (0.56, 0.66)
1	drinking 3-7 days/week	TC	85	19	0.95 (0.91, 1.00)	0.89 (0.83, 0.96)	0.88 (0.82, 0.95)	0.78 (0.69, 0.87)
1	drinking 3-7 days/week	FEC	75	32	0.73 (0.64, 0.84)	0.65 (0.55, 0.77)	0.63 (0.53, 0.75)	0.57 (0.47, 0.70)
2	never drinking	TC	294	77	0.93 (0.90, 0.96)	0.86 (0.82, 0.90)	0.84 (0.80, 0.88)	0.74 (0.69, 0.79)
2	never drinking	FEC	317	138	0.74 (0.69, 0.79)	0.66 (0.61, 0.72)	0.62 (0.57, 0.68)	0.56 (0.51, 0.62)
2	drinking 1-2 days/week	TC	291	71	0.91 (0.88, 0.94)	0.85 (0.81, 0.89)	0.81 (0.77, 0.86)	0.75 (0.71, 0.81)
2	drinking 1-2 days/week	FEC	322	120	0.75 (0.70, 0.80)	0.70 (0.65, 0.75)	0.66 (0.61, 0.71)	0.63 (0.58, 0.68)
2	drinking 3-7 days/week	TC	85	15	0.95 (0.91, 1.00)	0.86 (0.79, 0.94)	0.85 (0.77, 0.93)	0.82 (0.75, 0.91)
2	drinking 3-7 days/week	FEC	76	26	0.89 (0.83, 0.97)	0.84 (0.76, 0.93)	0.78 (0.69, 0.88)	0.66 (0.56, 0.77)
3	never drinking	TC	292	76	0.92 (0.89, 0.95)	0.86 (0.82, 0.90)	0.83 (0.79, 0.88)	0.74 (0.69, 0.79)
3	never drinking	FEC	310	122	0.74 (0.69, 0.79)	0.68 (0.63, 0.74)	0.65 (0.60, 0.71)	0.60 (0.55, 0.66)
3	drinking 1-2 days/week	TC	290	71	0.93 (0.91, 0.96)	0.88 (0.84, 0.92)	0.82 (0.78, 0.87)	0.75 (0.70, 0.80)
3	drinking 1-2 days/week	FEC	318	129	0.72 (0.67, 0.77)	0.67 (0.62, 0.72)	0.64 (0.59, 0.69)	0.59 (0.54, 0.65)
3	drinking 3-7 days/week	TC	85	14	0.93 (0.88, 0.99)	0.91 (0.85, 0.97)	0.87 (0.80, 0.94)	0.83 (0.76, 0.92)
3	drinking 3-7 days/week	FEC	75	23	0.87 (0.79, 0.95)	0.84 (0.76, 0.93)	0.80 (0.71, 0.90)	0.69 (0.59, 0.80)

Supplemental table 8. Emetic-event-free survival rates relative to cycle and BMI subgroup.

Cycle	BMI	Therapy	Patients	Events	12-hour emesis-free rate (95% CI)	24-hour emesis-free rate (95% CI)	48-hour emesis-free rate (95% CI)	120-hour emesis-free rate (95% CI)
1	underweight/normal	TC	333	87	0.93 (0.90, 0.96)	0.86 (0.82, 0.90)	0.84 (0.80, 0.88)	0.74 (0.69, 0.79)
1	underweight/normal	FEC	354	157	0.69 (0.65, 0.74)	0.64 (0.59, 0.69)	0.60 (0.55, 0.65)	0.55 (0.51, 0.61)
1	overweight	TC	244	62	0.91 (0.87, 0.94)	0.85 (0.81, 0.90)	0.83 (0.78, 0.88)	0.75 (0.69, 0.80)
1	overweight	FEC	253	107	0.72 (0.66, 0.77)	0.67 (0.62, 0.73)	0.64 (0.58, 0.70)	0.58 (0.52, 0.64)
1	obese	TC	154	51	0.86 (0.80, 0.91)	0.81 (0.75, 0.87)	0.77 (0.71, 0.84)	0.67 (0.60, 0.75)
1	obese	FEC	153	72	0.69 (0.62, 0.77)	0.61 (0.54, 0.70)	0.57 (0.49, 0.65)	0.53 (0.45, 0.61)
2	underweight/normal	TC	331	80	0.92 (0.90, 0.95)	0.84 (0.80, 0.88)	0.82 (0.78, 0.86)	0.76 (0.71, 0.81)
2	underweight/normal	FEC	354	142	0.74 (0.69, 0.78)	0.68 (0.63, 0.73)	0.64 (0.59, 0.69)	0.60 (0.55, 0.65)
2	overweight	TC	242	57	0.94 (0.91, 0.97)	0.87 (0.83, 0.91)	0.84 (0.80, 0.89)	0.76 (0.71, 0.82)
2	overweight	FEC	252	99	0.78 (0.73, 0.83)	0.73 (0.67, 0.78)	0.68 (0.62, 0.74)	0.61 (0.55, 0.67)
2	obese	TC	152	43	0.91 (0.87, 0.96)	0.85 (0.80, 0.91)	0.81 (0.75, 0.88)	0.71 (0.65, 0.79)
2	obese	FEC	153	64	0.75 (0.68, 0.82)	0.67 (0.60, 0.75)	0.63 (0.55, 0.71)	0.58 (0.51, 0.66)
3	underweight/normal	TC	330	82	0.93 (0.90, 0.96)	0.86 (0.82, 0.90)	0.81 (0.76, 0.85)	0.75 (0.70, 0.80)
3	underweight/normal	FEC	349	144	0.71 (0.66, 0.76)	0.66 (0.61, 0.71)	0.63 (0.58, 0.68)	0.59 (0.54, 0.64)
3	overweight	TC	242	58	0.94 (0.91, 0.97)	0.90 (0.86, 0.94)	0.86 (0.82, 0.91)	0.76 (0.71, 0.81)
3	overweight	FEC	249	93	0.77 (0.72, 0.83)	0.71 (0.66, 0.77)	0.69 (0.64, 0.75)	0.63 (0.57, 0.69)
3	obese	TC	152	38	0.93 (0.89, 0.97)	0.87 (0.82, 0.92)	0.84 (0.78, 0.90)	0.75 (0.68, 0.82)
3	obese	FEC	148	54	0.76 (0.69, 0.83)	0.72 (0.65, 0.80)	0.68 (0.61, 0.76)	0.63 (0.56, 0.72)

Supplemental figure 1. Nausea-free survival described via Kaplan Meier curves between the FEC and the TC group for age subgroups. A shows cycle 1, B cycle 2, and C cycle 3.

A

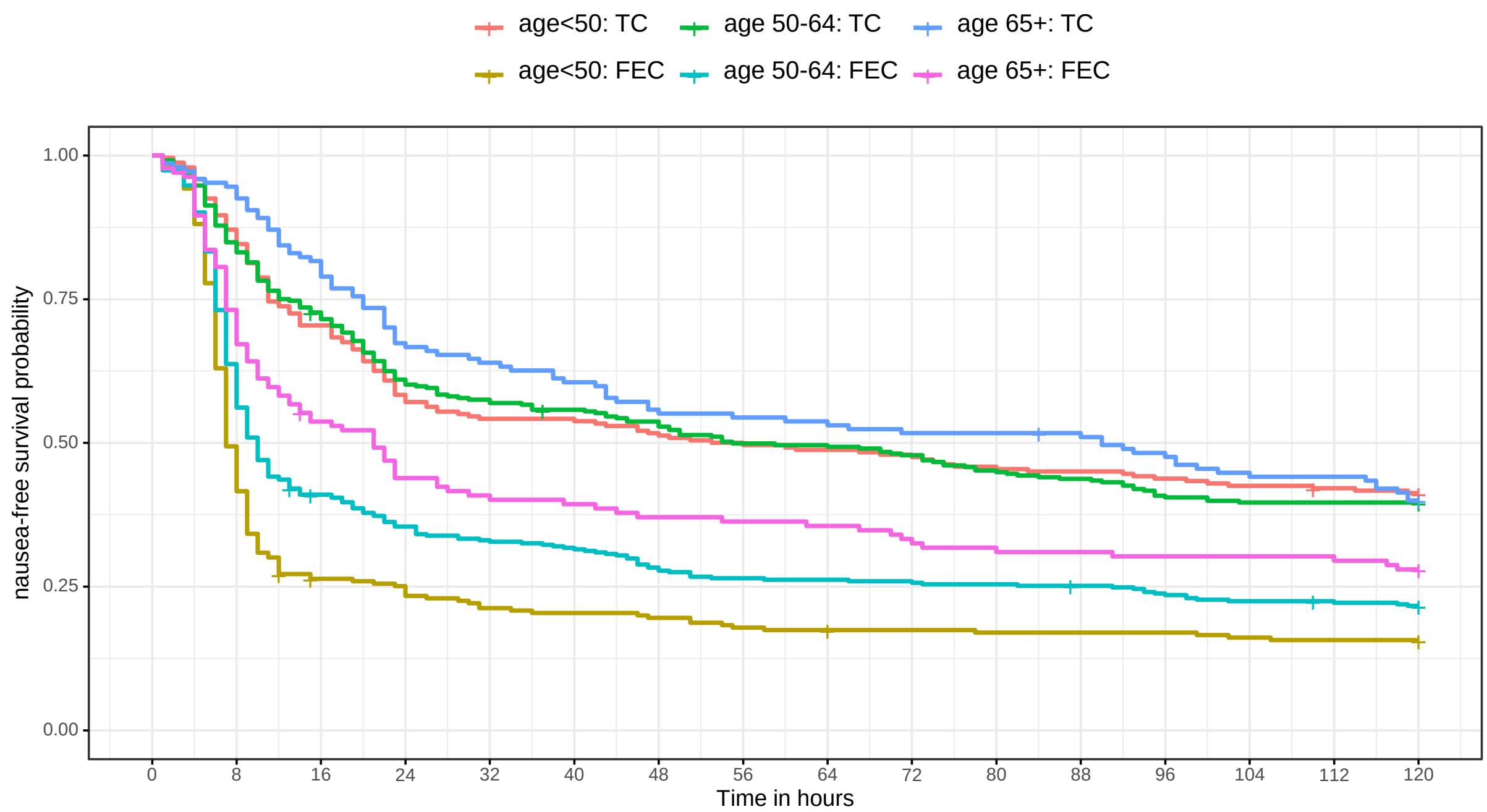

Number at risk

	0	8	16	24	32	40	48	56	64	72	80	88	96	104	112	120
age<50: TC	240	209	169	140	130	130	124	120	117	115	110	108	105	102	100	98
age<50: FEC	243	120	62	59	50	48	46	42	41	40	39	39	39	37	36	36
age 50-64: TC	344	292	249	209	197	190	183	170	169	163	154	149	139	135	135	135
age 50-64: FEC	383	244	155	134	125	120	107	100	99	98	96	94	89	84	83	80
age 65+: TC	147	139	120	99	94	89	82	80	79	76	76	75	70	65	64	58
age 65+: FEC	134	98	71	58	54	52	49	48	47	44	42	41	40	40	40	37

Time in hours

B

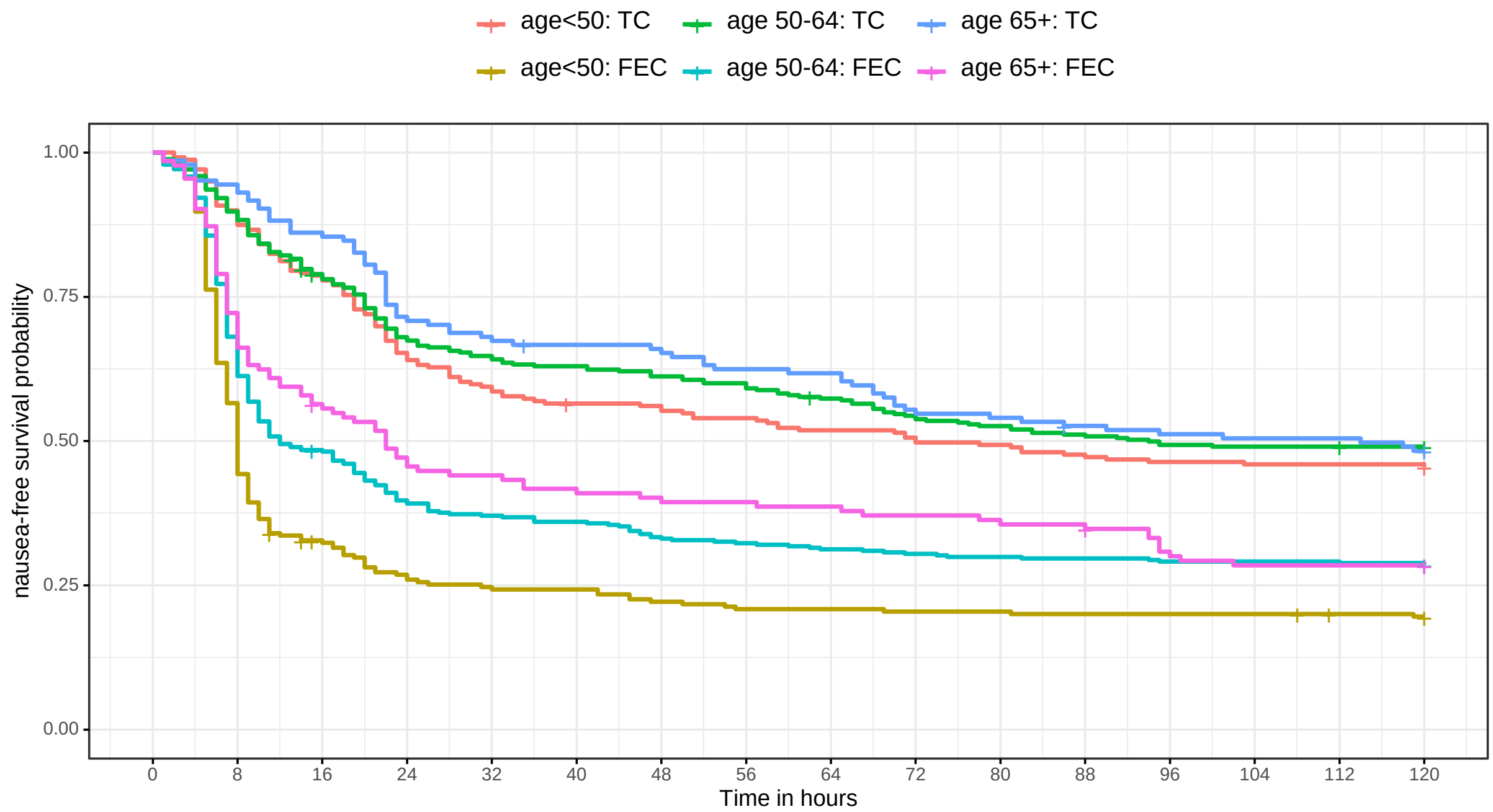

Number at risk

	0	8	16	24	32	40	48	56	64	72	80	88	96	104	112	120
age<50: TC	239	215	188	156	142	134	133	128	123	120	117	113	110	109	109	109
age<50: FEC	244	138	77	63	58	57	52	49	49	48	48	47	47	47	45	44
age 50-64: TC	342	307	267	230	219	213	207	203	193	183	177	172	166	165	165	164
age 50-64: FEC	382	260	183	150	140	136	126	122	118	115	113	112	110	110	110	109
age 65+: TC	144	136	124	103	98	95	94	89	88	79	77	73	71	70	70	67
age 65+: FEC	133	96	73	61	57	54	52	51	50	48	47	46	39	36	36	36

Time in hours

C

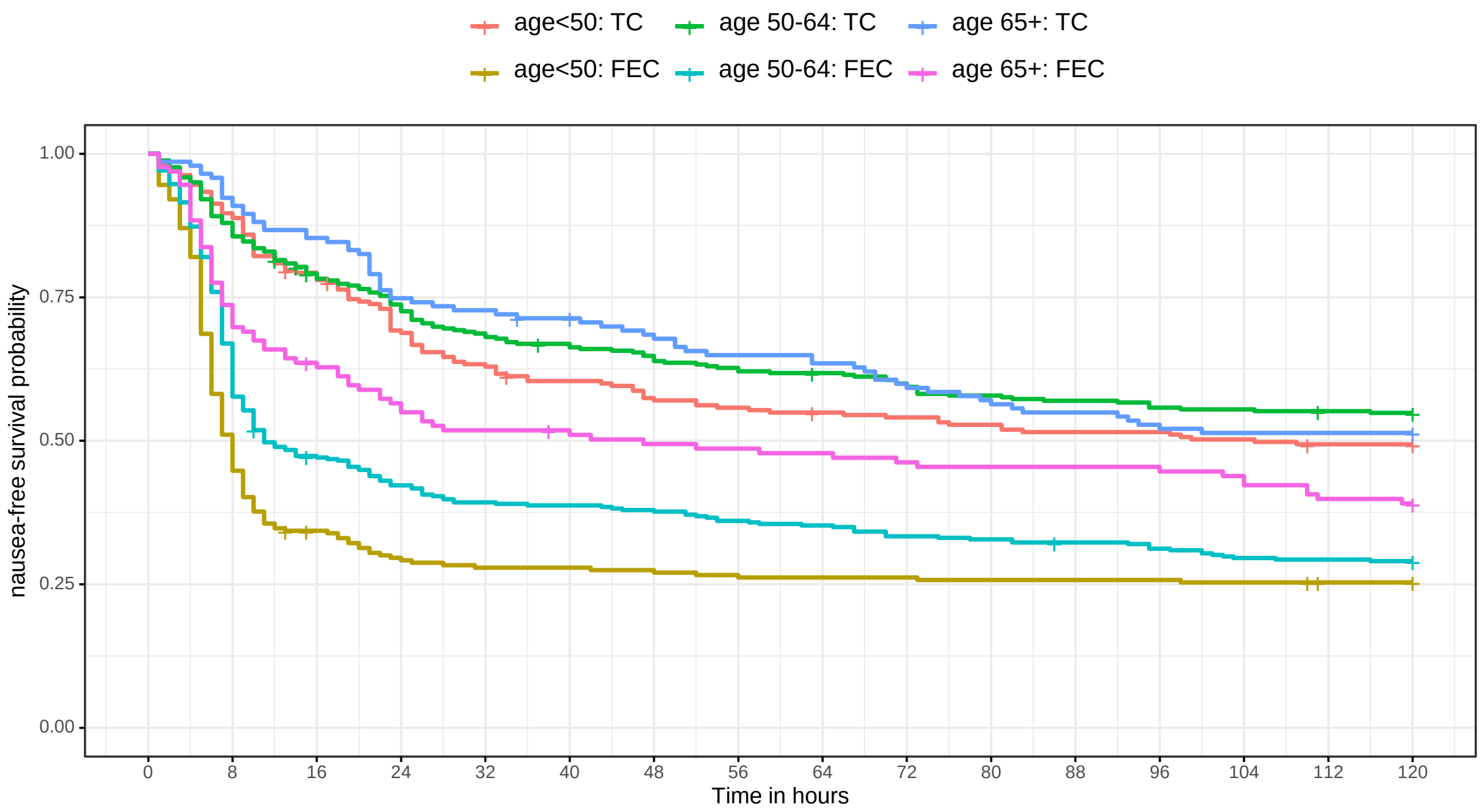

Number at risk

	0	8	16	24	32	40	48	56	64	72	80	88	96	104	112	120
age<50: TC	241	216	190	165	151	143	136	132	129	127	124	121	121	118	115	115
age<50: FEC	239	122	80	69	65	65	64	62	61	61	60	60	60	59	57	57
age 50-64: TC	340	299	265	247	230	223	216	209	205	199	192	189	185	184	182	181
age 50-64: FEC	378	253	176	157	146	144	141	134	131	124	122	119	115	109	108	107
age 65+: TC	143	132	122	107	104	101	96	91	89	84	80	77	74	72	72	72
age 65+: FEC	129	95	81	72	66	65	62	61	60	58	57	57	57	55	50	49

Time in hours

Supplemental figure 2. Nausea-free survival described via Kaplan Meier curves between the FEC and the TC group for smoking subgroups. A shows cycle 1, B cycle 2, and C cycle 3.

A

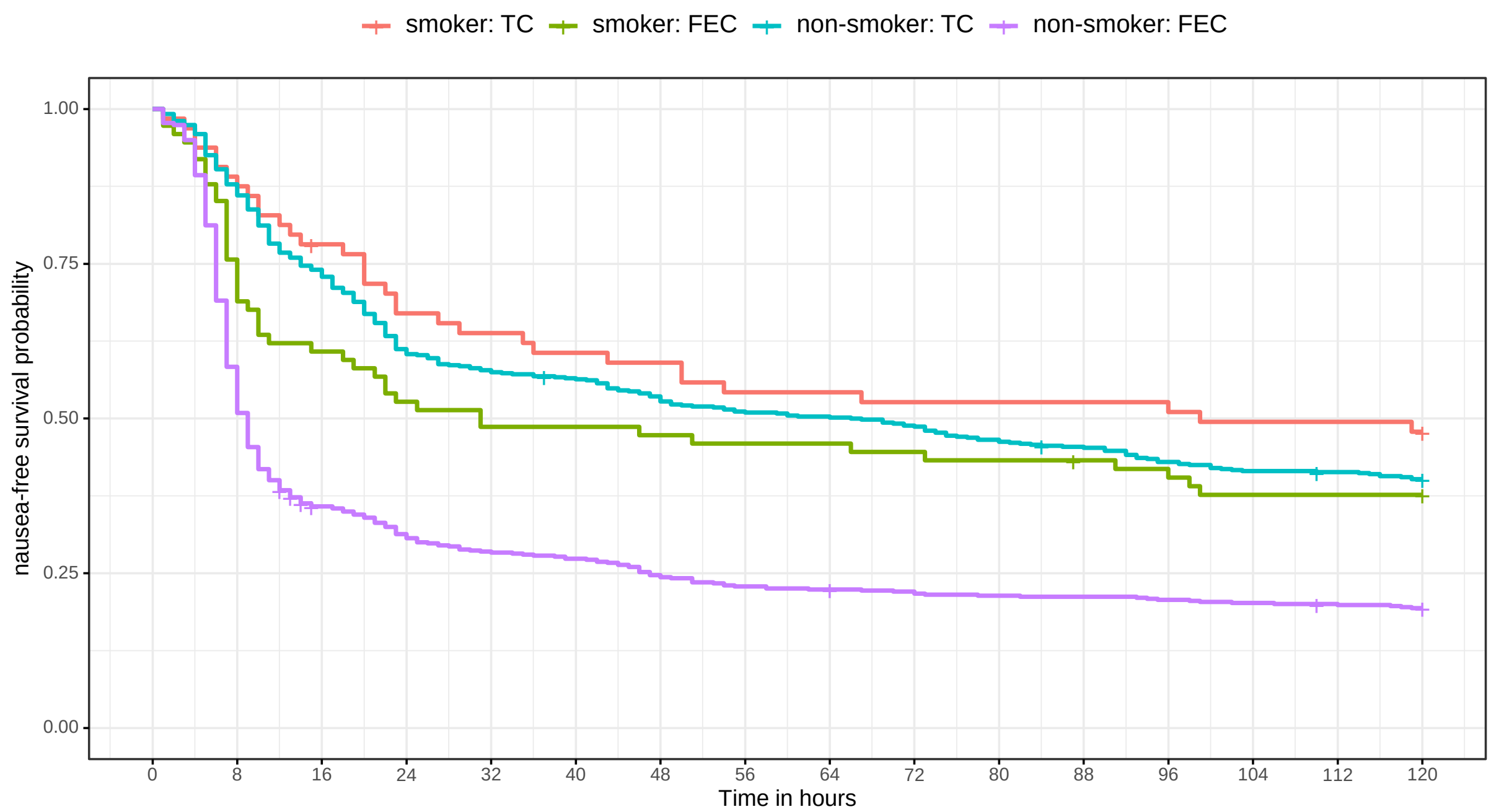

Number at risk

	0	8	16	24	32	40	48	56	64	72	80	88	96	104	112	120
smoker: TC	64	57	49	42	40	38	37	34	34	33	33	33	33	31	31	30
smoker: FEC	74	56	45	39	36	36	35	34	34	33	32	31	30	27	27	27
non-smoker: TC	616	541	456	377	356	347	329	314	309	300	286	278	263	254	252	245
non-smoker: FEC	617	360	216	189	172	165	149	138	135	132	128	127	124	121	119	115

Time in hours

B

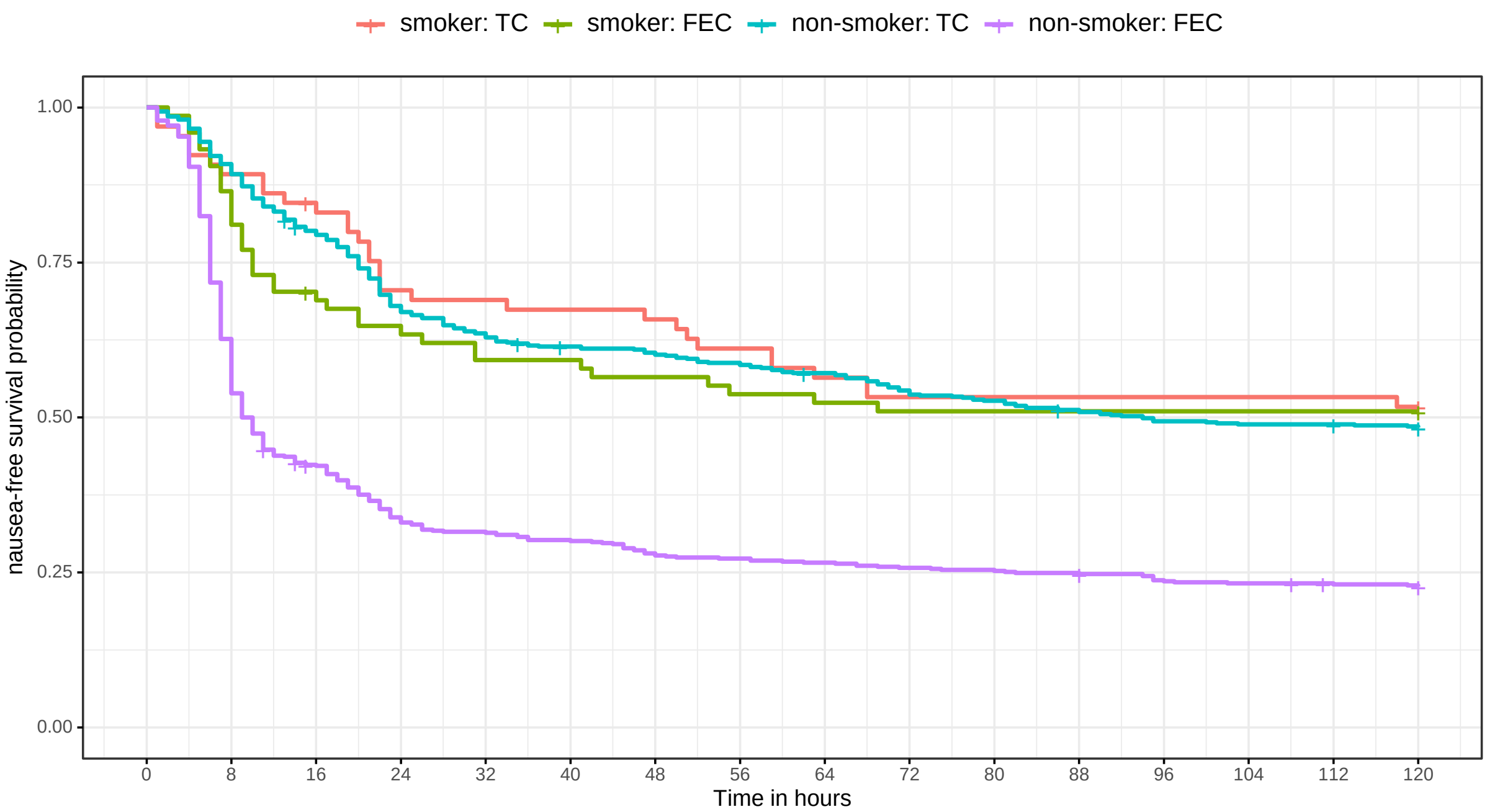

Number at risk

	0	8	16	24	32	40	48	56	64	72	80	88	96	104	112	120
smoker: TC	65	58	54	45	44	43	42	39	36	34	34	34	34	34	34	33
smoker: FEC	74	64	51	47	43	43	41	39	38	37	37	37	37	37	37	37
non-smoker: TC	613	557	489	415	388	373	367	357	346	329	319	308	297	294	294	291
non-smoker: FEC	616	386	255	204	190	182	169	164	160	155	153	150	142	139	137	135

Time in hours

C

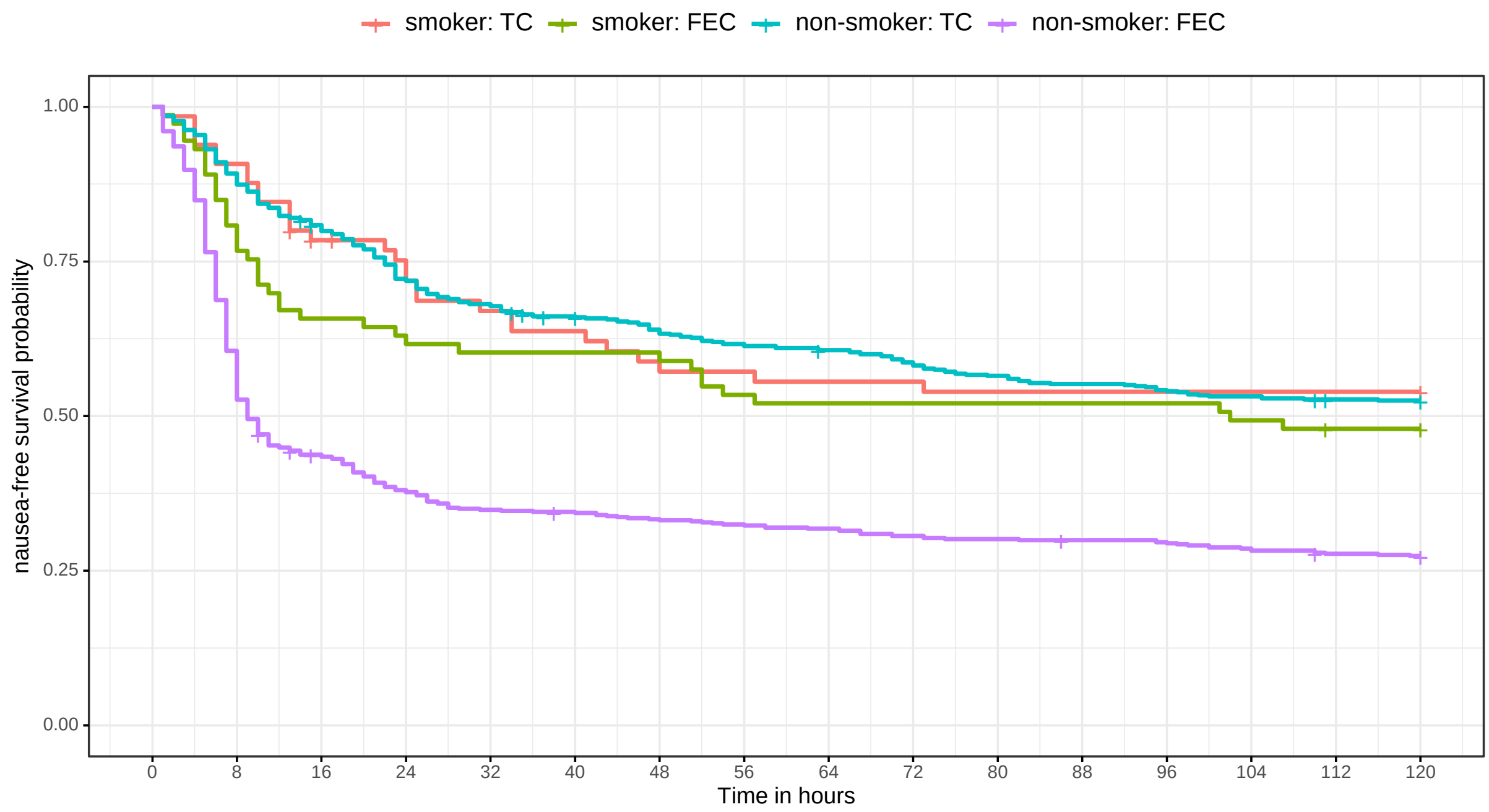

Number at risk

	0	8	16	24	32	40	48	56	64	72	80	88	96	104	112	120
smoker: TC	65	59	49	46	41	39	36	35	34	34	33	33	33	33	33	33
smoker: FEC	73	59	48	46	44	44	44	39	38	38	38	38	38	36	34	34
non-smoker: TC	612	546	493	440	415	400	386	372	365	353	340	332	326	320	315	314
non-smoker: FEC	608	368	260	226	207	204	197	192	188	181	178	176	174	168	162	160

Time in hours

Supplemental figure 3. Nausea-free survival described via Kaplan Meier curves between the FEC and the TC group for alcohol subgroups. A shows cycle 1, B cycle 2, and C cycle 3.

A

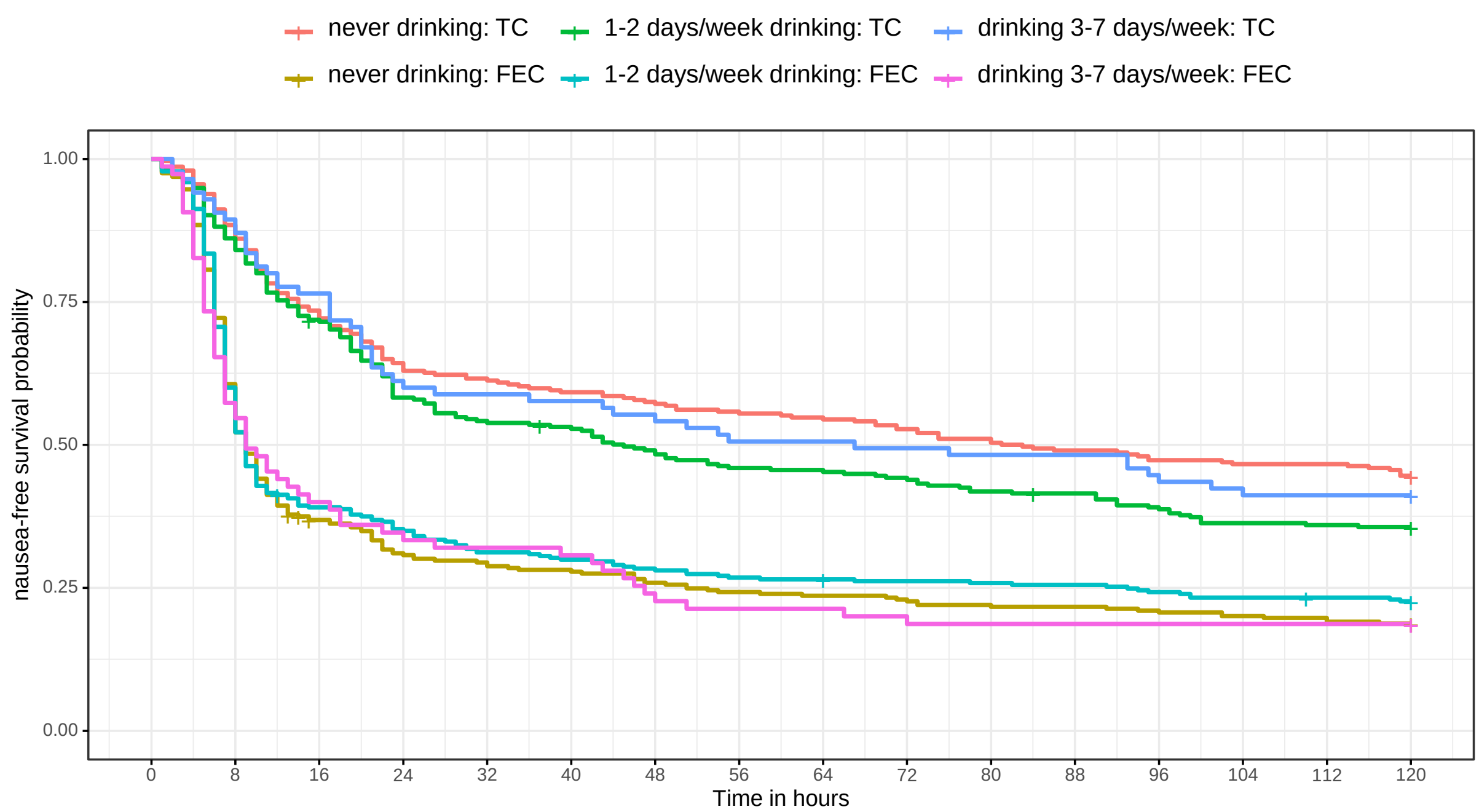

Number at risk

	0	8	16	24	32	40	48	56	64	72	80	88	96	104	112	120
never drinking: TC	294	260	216	189	181	174	169	164	161	155	150	144	139	137	137	131
never drinking: FEC	320	194	114	96	91	87	80	75	73	71	68	67	65	62	61	58
1-2 days/week drinking: TC	295	254	211	171	159	155	143	134	133	129	122	120	113	105	104	103
1-2 days/week drinking: FEC	320	192	124	112	99	95	90	85	84	82	81	80	76	73	72	70
drinking 3-7 days/week: TC	85	76	65	52	50	49	47	43	43	42	41	41	38	36	35	35
drinking 3-7 days/week: FEC	75	43	30	26	24	23	18	16	16	15	14	14	14	14	14	14

Time in hours

B

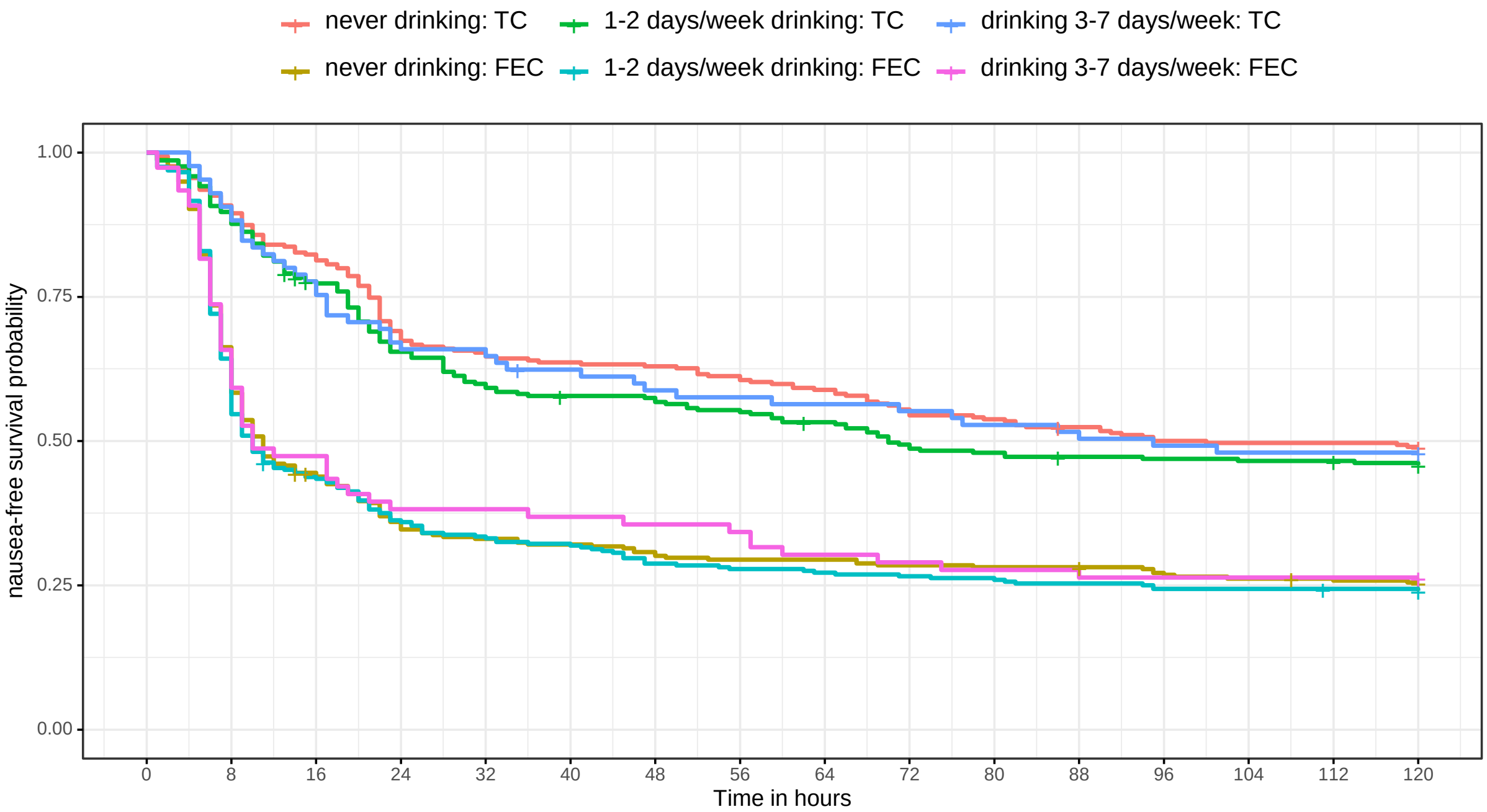

Number at risk

	0	8	16	24	32	40	48	56	64	72	80	88	96	104	112	120
never drinking: TC	294	267	242	203	192	187	185	180	173	163	158	153	146	145	145	143
never drinking: FEC	317	210	136	110	101	98	94	90	90	87	86	86	82	79	78	76
1-2 days/week drinking: TC	291	261	223	188	172	165	164	158	151	140	136	133	132	131	131	129
1-2 days/week drinking: FEC	322	207	140	116	107	103	92	89	87	85	84	81	78	78	77	77
drinking 3-7 days/week: TC	85	77	66	57	56	52	49	48	47	46	44	43	41	40	40	40
drinking 3-7 days/week: FEC	76	50	36	29	29	28	27	26	23	22	21	21	20	20	20	20

Time in hours

C

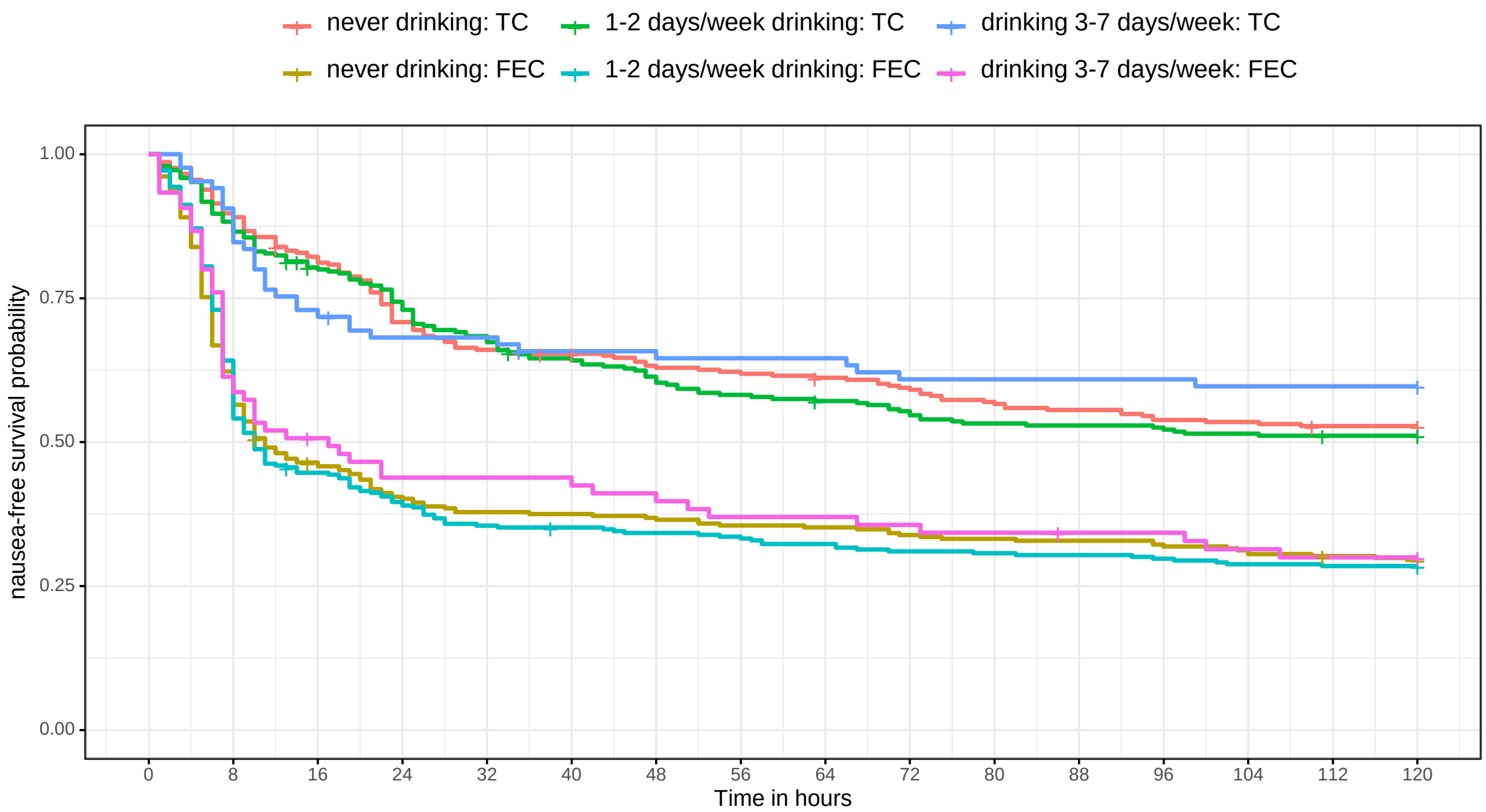

Number at risk

	0	8	16	24	32	40	48	56	64	72	80	88	96	104	112	120
never drinking: TC	292	262	239	206	192	189	182	179	175	170	163	159	154	153	150	150
never drinking: FEC	310	193	140	122	114	113	111	107	106	102	100	99	97	94	90	88
1-2 days/week drinking: TC	290	256	229	212	195	183	174	165	161	156	150	149	148	145	143	143
1-2 days/week drinking: FEC	318	204	141	125	112	110	107	105	101	97	96	95	93	90	89	89
drinking 3-7 days/week: TC	85	77	62	57	57	54	54	53	53	50	50	50	50	49	49	49
drinking 3-7 days/week: FEC	75	46	37	32	32	32	30	27	27	26	25	24	24	22	21	21

Time in hours

Supplemental figure 4. Nausea-free survival described via Kaplan Meier curves between the FEC and the TC group for BMI subgroups. A shows cycle 1, B cycle 2, and C cycle 3.

A

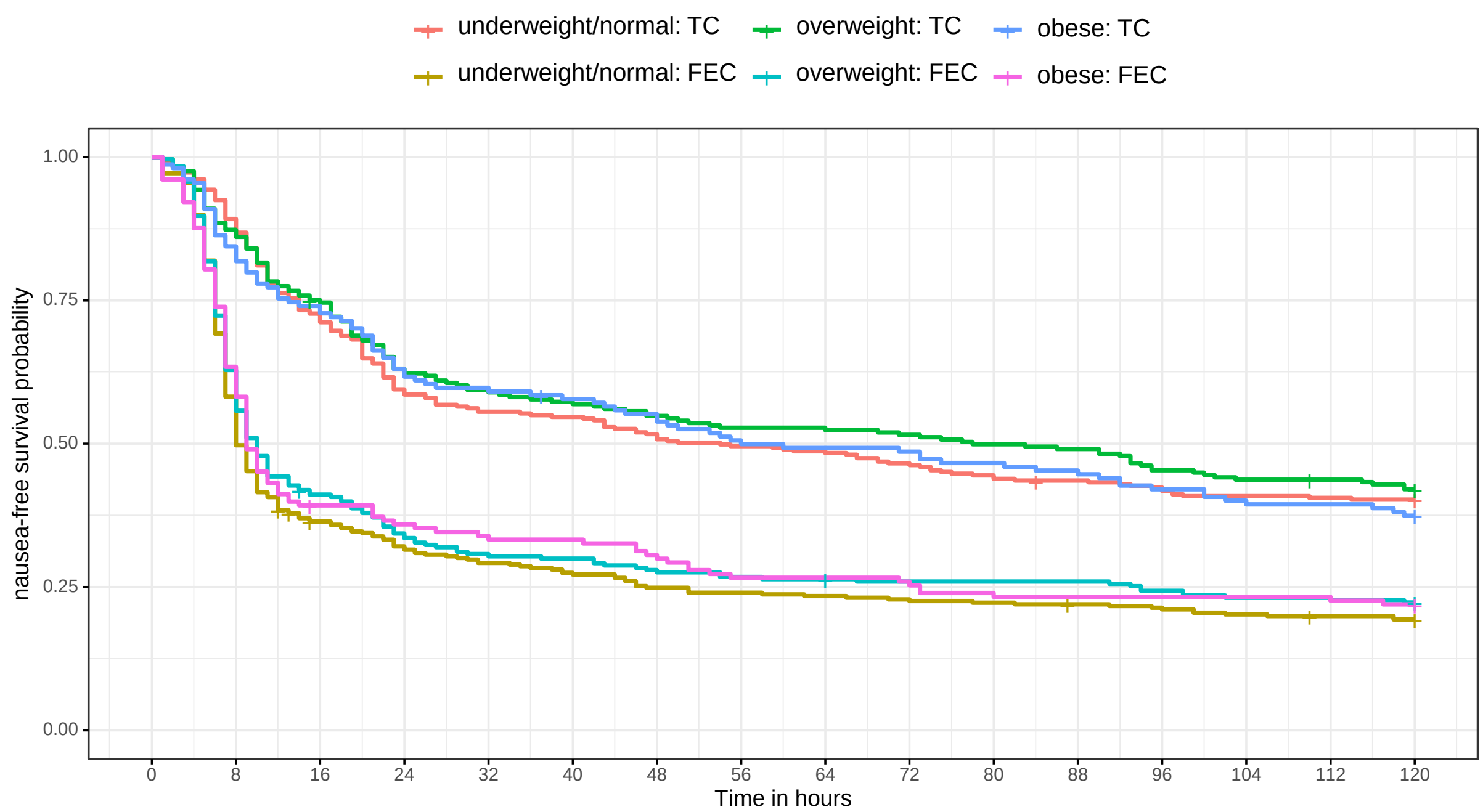

Number at risk

	0	8	16	24	32	40	48	56	64	72	80	88	96	104	112	120
underweight/normal: TC	333	297	242	198	185	182	172	165	162	155	148	144	140	135	134	133
underweight/normal: FEC	354	206	126	111	101	95	86	83	81	79	77	75	73	69	67	65
overweight: TC	244	213	182	153	144	139	133	128	128	125	121	119	110	106	105	101
overweight: FEC	253	159	103	86	77	75	70	67	66	64	64	64	60	57	57	55
obese: TC	154	130	114	97	92	88	84	77	75	74	71	69	64	61	60	57
obese: FEC	153	97	59	54	51	50	46	40	40	39	36	35	35	35	35	33

Time in hours

B

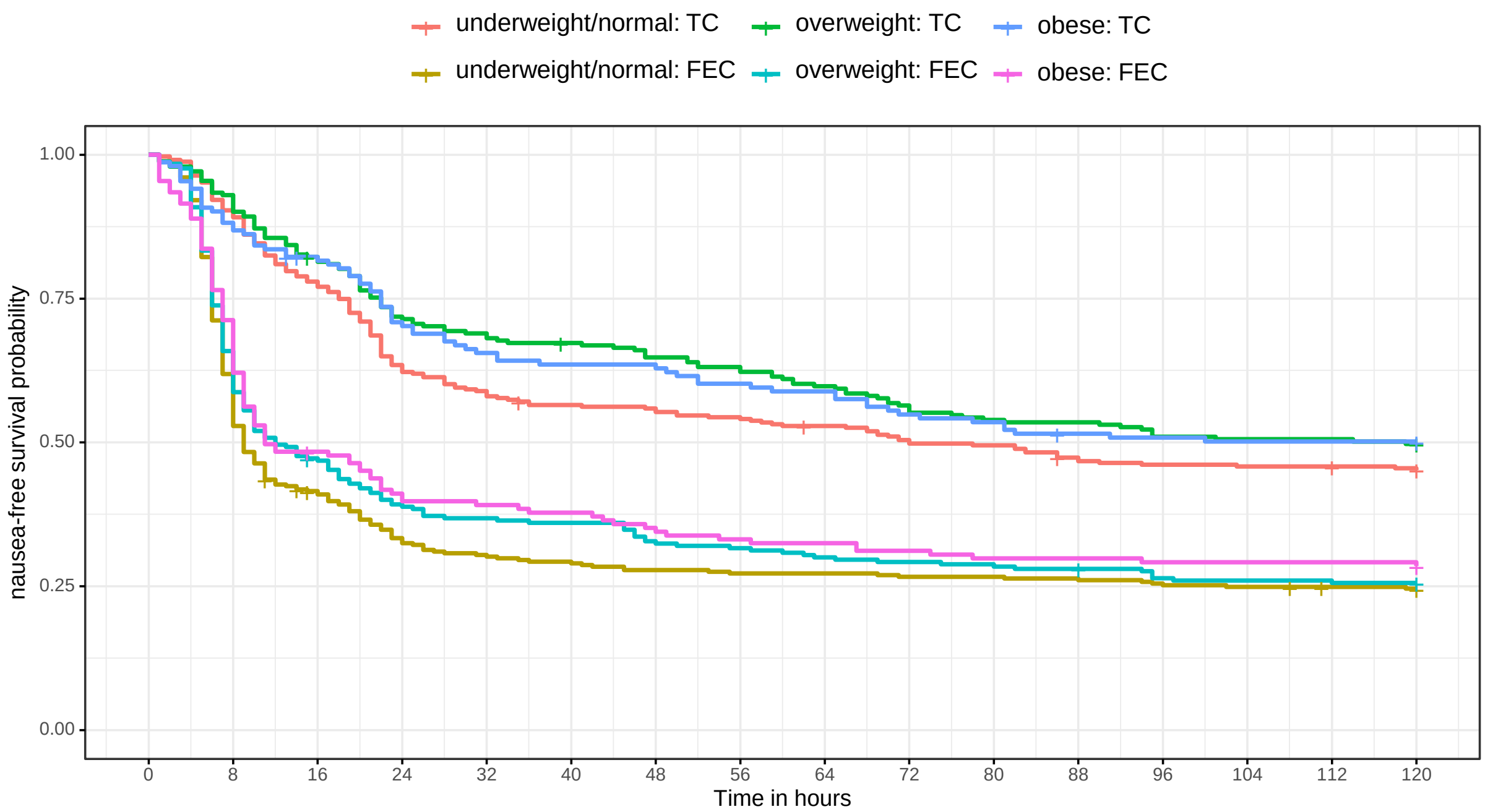

Number at risk

	0	8	16	24	32	40	48	56	64	72	80	88	96	104	112	120
underweight/normal: TC	331	299	258	210	195	186	184	179	173	165	162	154	150	149	149	147
underweight/normal: FEC	354	219	142	114	104	100	95	93	93	91	91	90	87	85	83	82
overweight: TC	242	225	198	173	166	161	155	151	143	135	129	128	122	121	121	119
overweight: FEC	252	166	118	98	92	90	82	79	75	73	72	70	65	64	64	63
obese: TC	152	134	123	106	98	95	95	90	88	82	80	76	75	74	74	74
obese: FEC	153	109	73	62	59	57	53	50	49	47	45	45	44	44	44	44

Time in hours

C

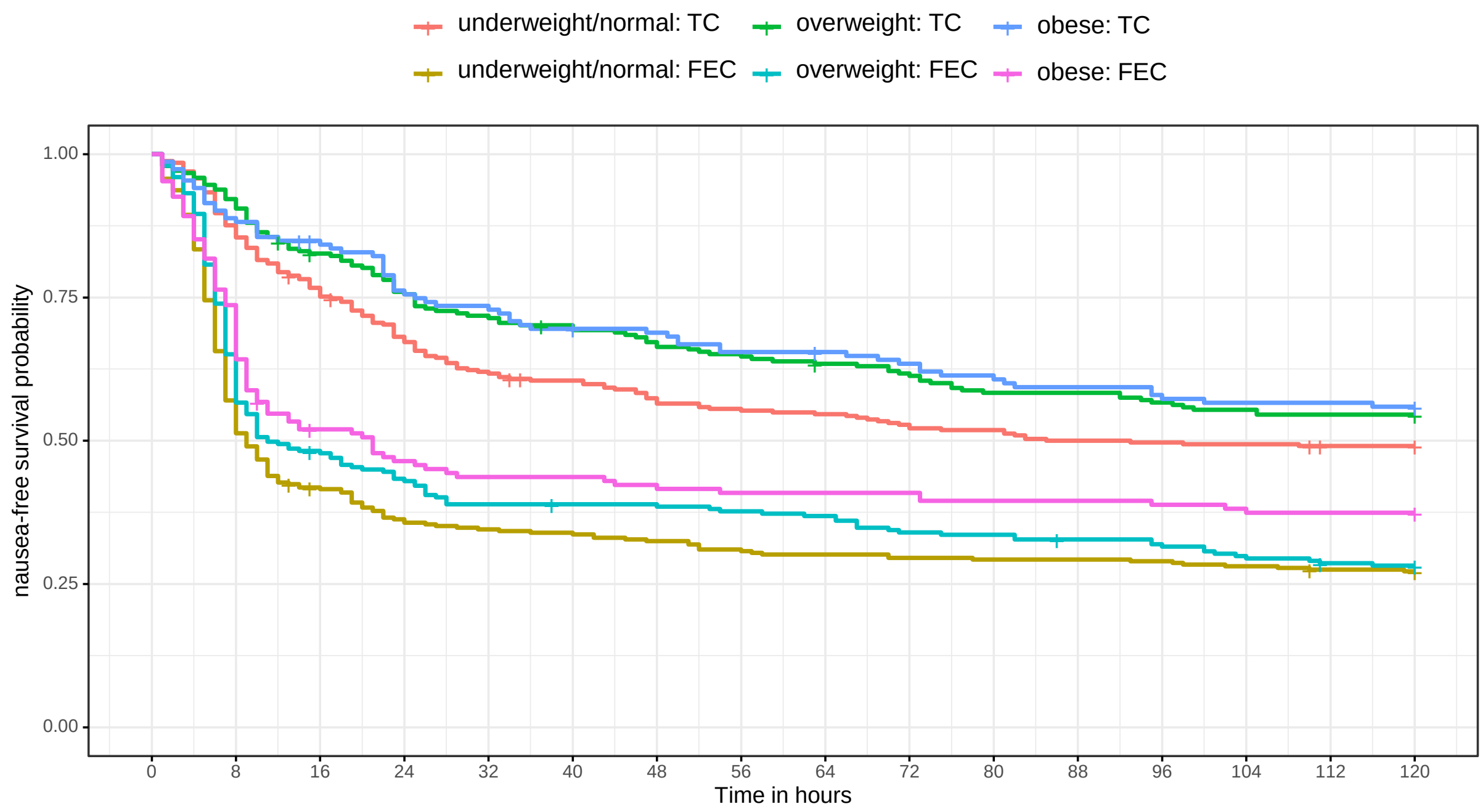

Number at risk

	0	8	16	24	32	40	48	56	64	72	80	88	96	104	112	120
underweight/normal: TC	330	289	252	223	203	196	186	180	177	171	168	162	161	160	157	157
underweight/normal: FEC	349	199	143	124	118	116	111	106	103	101	100	100	99	96	93	92
overweight: TC	242	223	198	182	172	167	160	155	150	146	138	138	134	131	129	129
overweight: FEC	249	162	119	107	96	95	95	92	90	83	82	79	77	72	68	67
obese: TC	152	135	127	114	110	104	102	97	96	93	90	87	85	83	83	82
obese: FEC	148	109	75	67	63	63	61	59	59	59	57	57	56	55	54	54

Time in hours

Supplemental figure 5. Emesis-free survival described via Kaplan Meier curves between the FEC and the TC group for age subgroups. A shows cycle 1, B cycle 2, and C cycle 3.

A

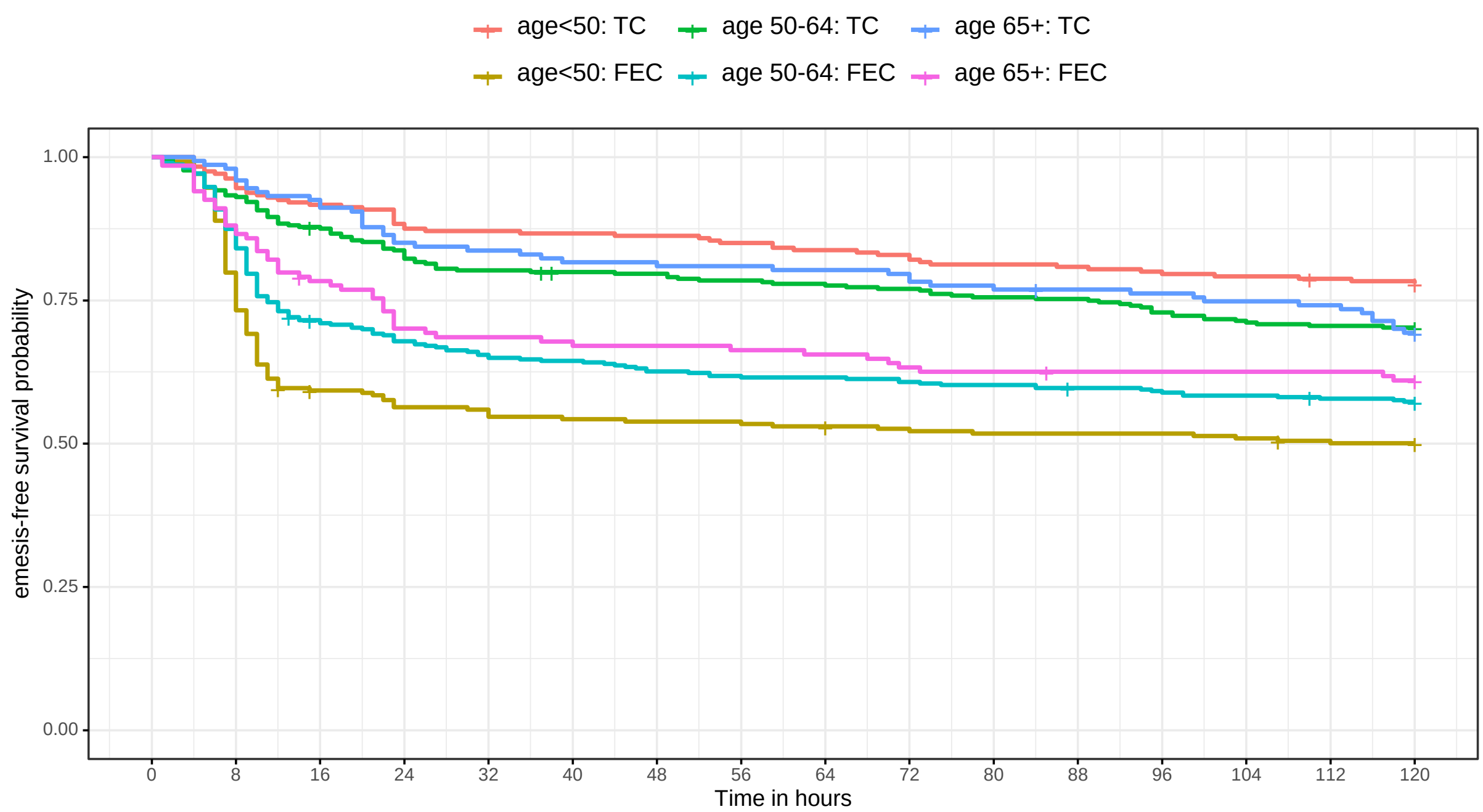

Number at risk

	0	8	16	24	32	40	48	56	64	72	80	88	96	104	112	120
age<50: TC	240	231	220	212	209	208	207	204	201	199	195	194	192	190	188	187
age<50: FEC	243	194	142	135	134	130	129	129	127	125	123	123	123	121	119	118
age 50-64: TC	344	321	301	287	275	272	271	267	265	262	257	256	248	243	240	239
age 50-64: FEC	383	335	272	258	249	245	238	235	234	231	229	225	223	220	217	215
age 65+: TC	147	144	136	125	123	120	120	119	118	117	114	112	111	109	108	101
age 65+: FEC	134	118	104	93	91	90	89	88	87	84	83	82	82	82	82	80

Time in hours

B

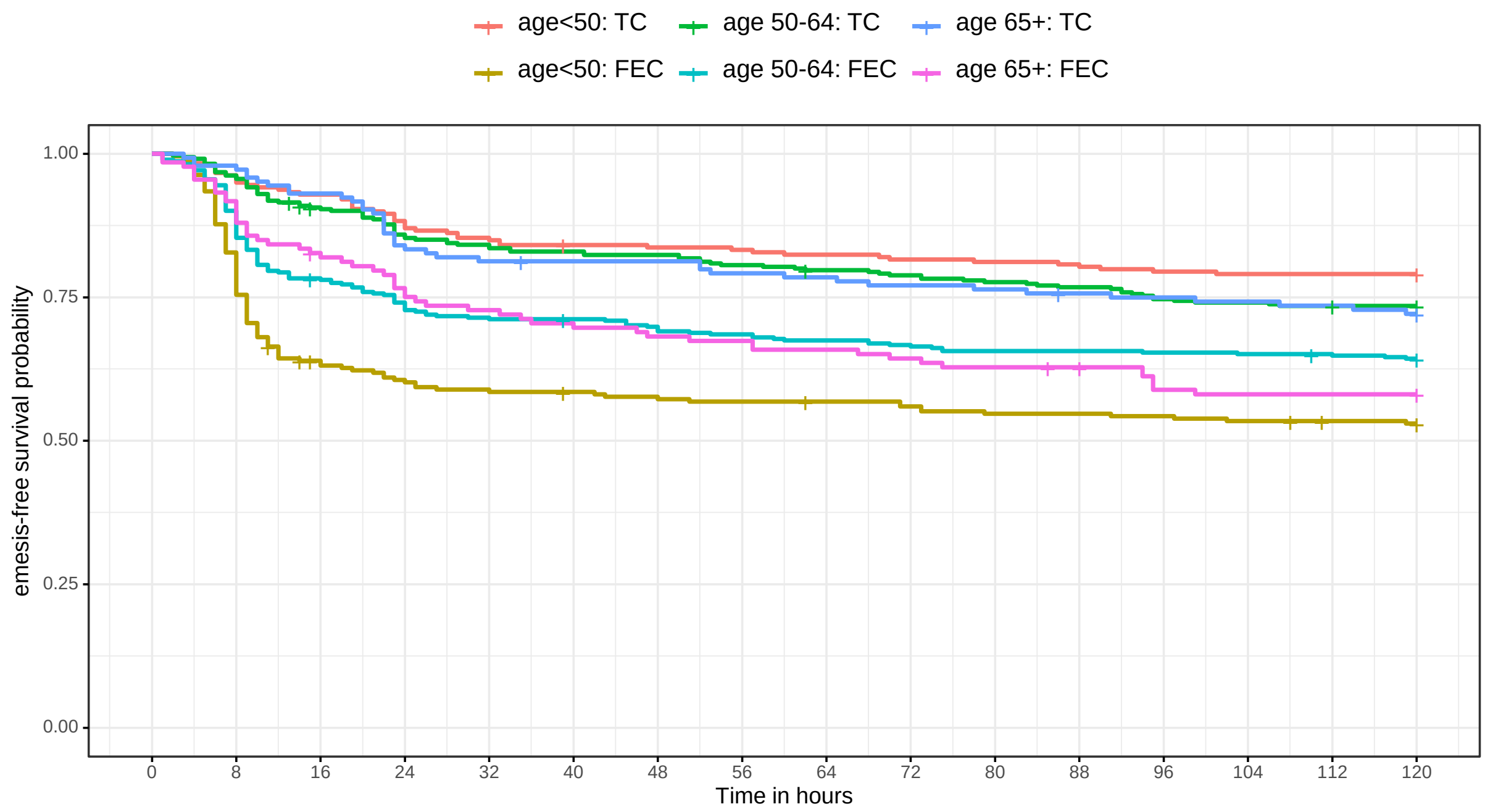

Number at risk

	0	8	16	24	32	40	48	56	64	72	80	88	96	104	112	120
age<50: TC	239	230	222	211	204	200	199	198	196	194	193	192	189	188	188	188
age<50: FEC	244	202	153	145	141	139	137	135	134	132	129	129	128	126	124	123
age 50-64: TC	342	329	307	291	285	281	279	273	269	266	262	259	252	250	248	247
age 50-64: FEC	382	344	297	281	271	269	264	259	255	252	248	248	247	246	245	242
age 65+: TC	144	141	134	121	117	116	116	113	112	110	109	106	105	104	103	101
age 65+: FEC	133	122	108	100	95	92	89	88	86	84	82	81	75	74	74	74

Time in hours

C

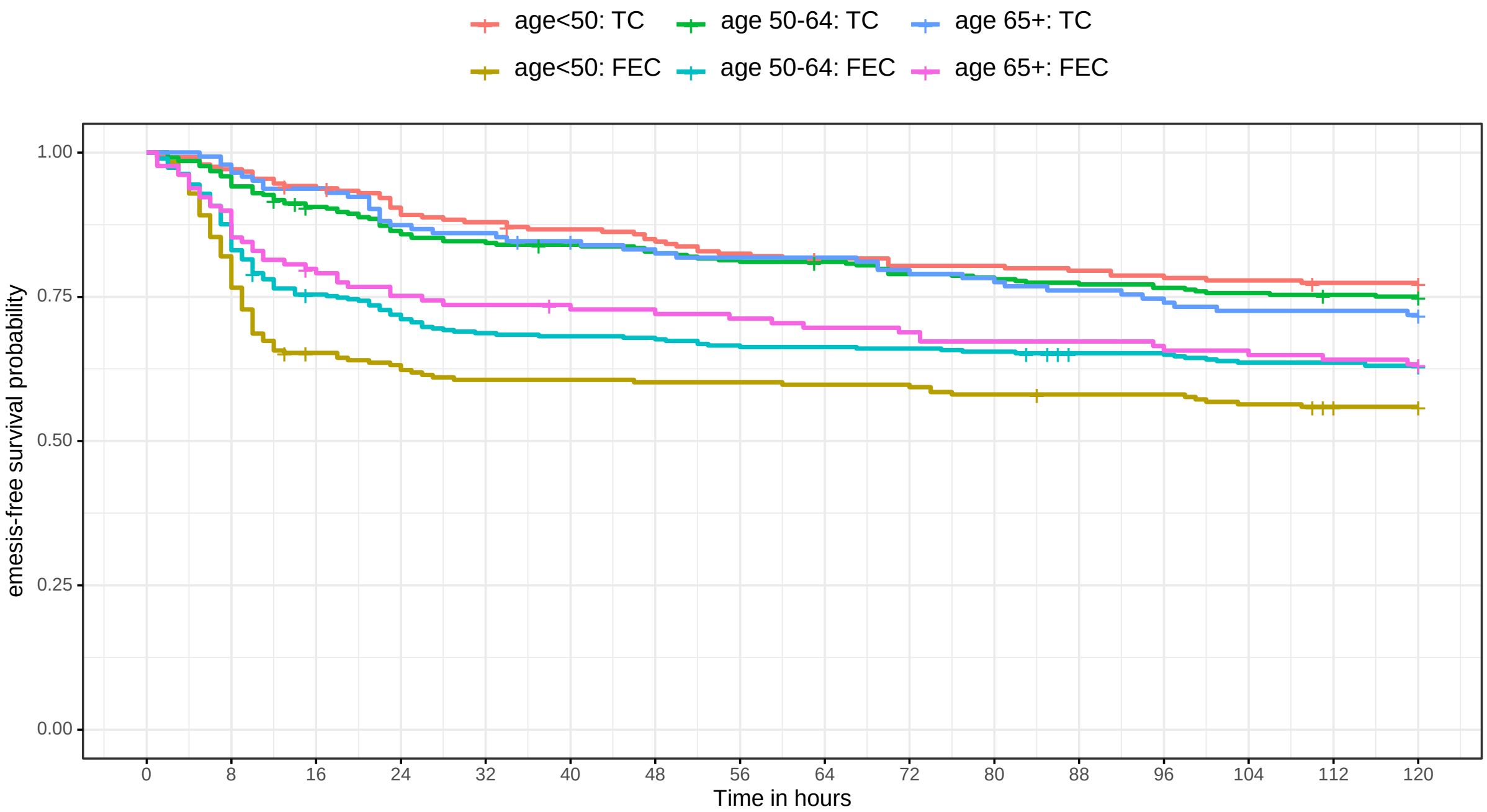

Number at risk

	0	8	16	24	32	40	48	56	64	72	80	88	96	104	112	120
age<50: TC	241	234	226	216	210	206	202	196	193	190	190	188	186	184	182	182
age<50: FEC	239	196	154	149	143	143	142	142	141	141	137	136	136	132	129	128
age 50-64: TC	340	326	304	290	284	281	277	272	270	263	261	258	255	252	250	249
age 50-64: FEC	378	331	282	269	257	255	254	249	248	247	245	240	240	234	234	232
age 65+: TC	143	140	134	125	123	120	117	115	115	112	110	107	105	102	102	101
age 65+: FEC	129	116	102	96	94	93	92	90	88	87	85	85	84	83	81	80

Time in hours

Supplemental figure 6. Emesis-free survival described via Kaplan Meier curves between the FEC and the TC group for smoking subgroups. A shows cycle 1, B cycle 2, and C cycle 3.

A

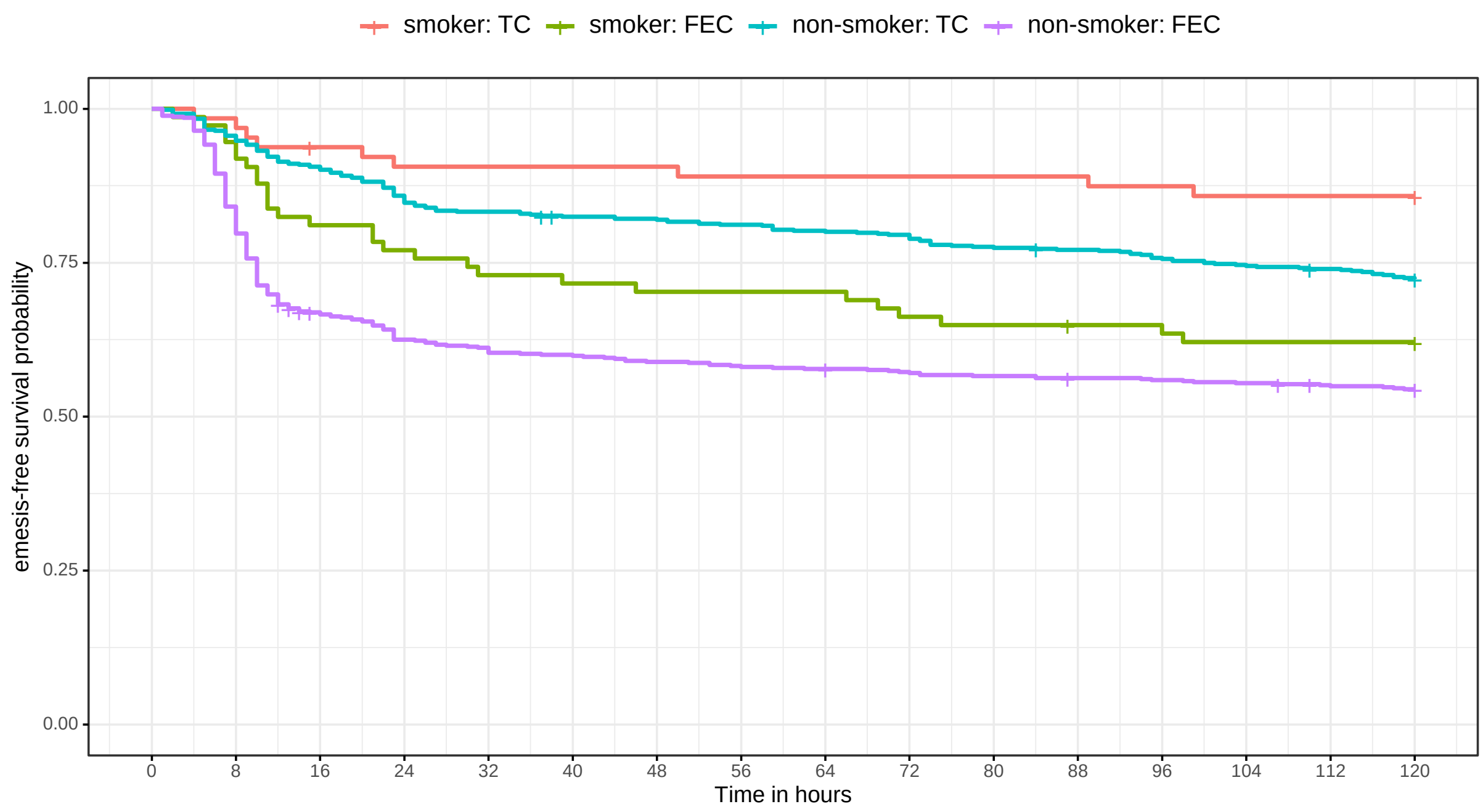

Number at risk

	0	8	16	24	32	40	48	56	64	72	80	88	96	104	112	120
smoker: TC	64	63	59	57	57	57	57	56	56	56	56	56	55	54	54	54
smoker: FEC	74	70	60	57	54	53	52	52	52	49	48	47	47	45	45	45
non-smoker: TC	616	589	558	529	513	506	504	498	492	488	476	472	464	457	452	443
non-smoker: FEC	617	519	408	381	373	366	359	355	352	348	344	341	339	336	332	328

Time in hours

B

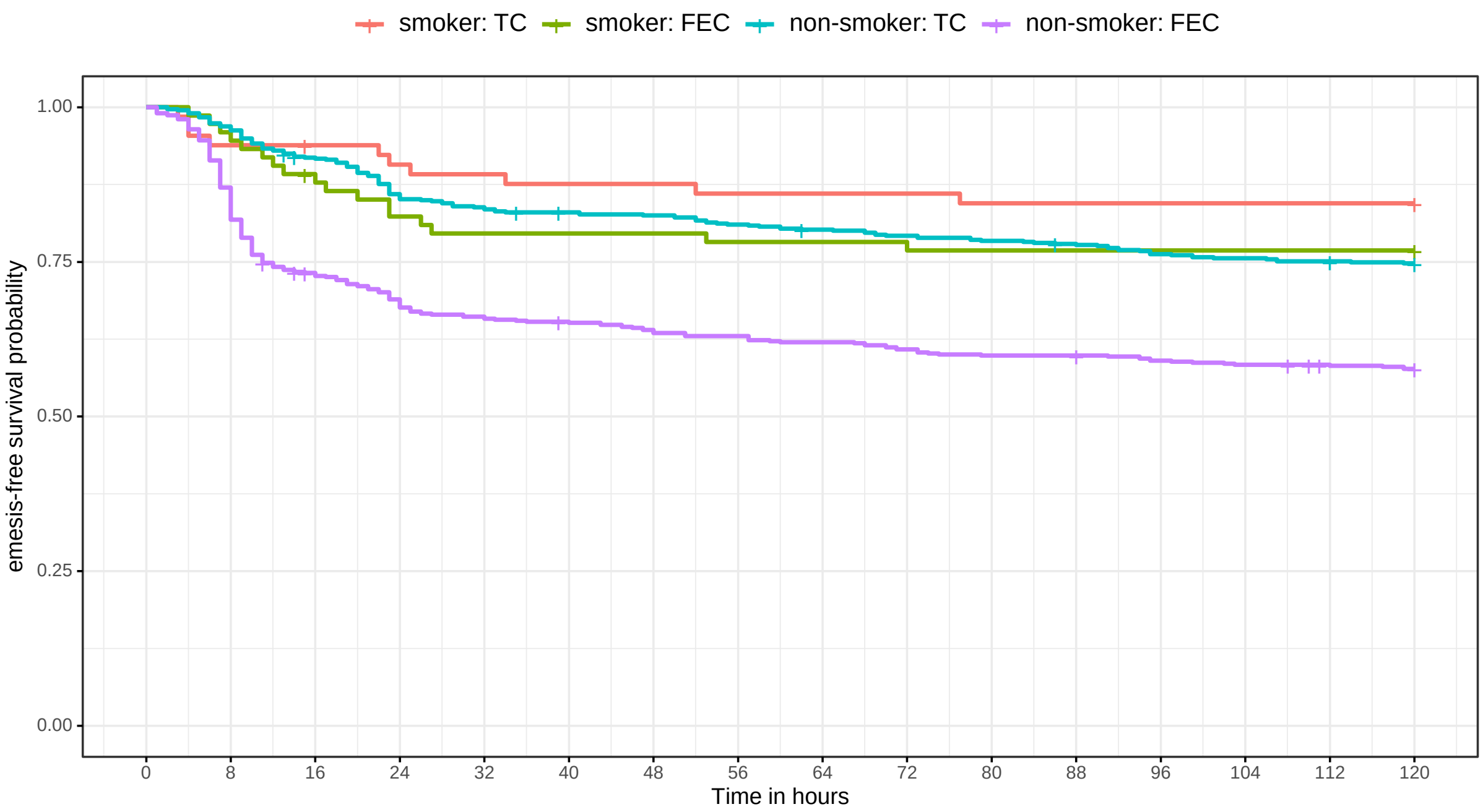

Number at risk

	0	8	16	24	32	40	48	56	64	72	80	88	96	104	112	120
smoker: TC	65	61	60	58	57	56	56	55	55	55	54	54	54	54	54	54
smoker: FEC	74	71	65	60	58	58	58	57	57	57	56	56	56	56	56	56
non-smoker: TC	613	594	561	525	512	505	502	493	487	481	476	471	461	457	454	451
non-smoker: FEC	616	536	445	419	402	395	387	381	375	368	362	362	356	352	349	345

Time in hours

C

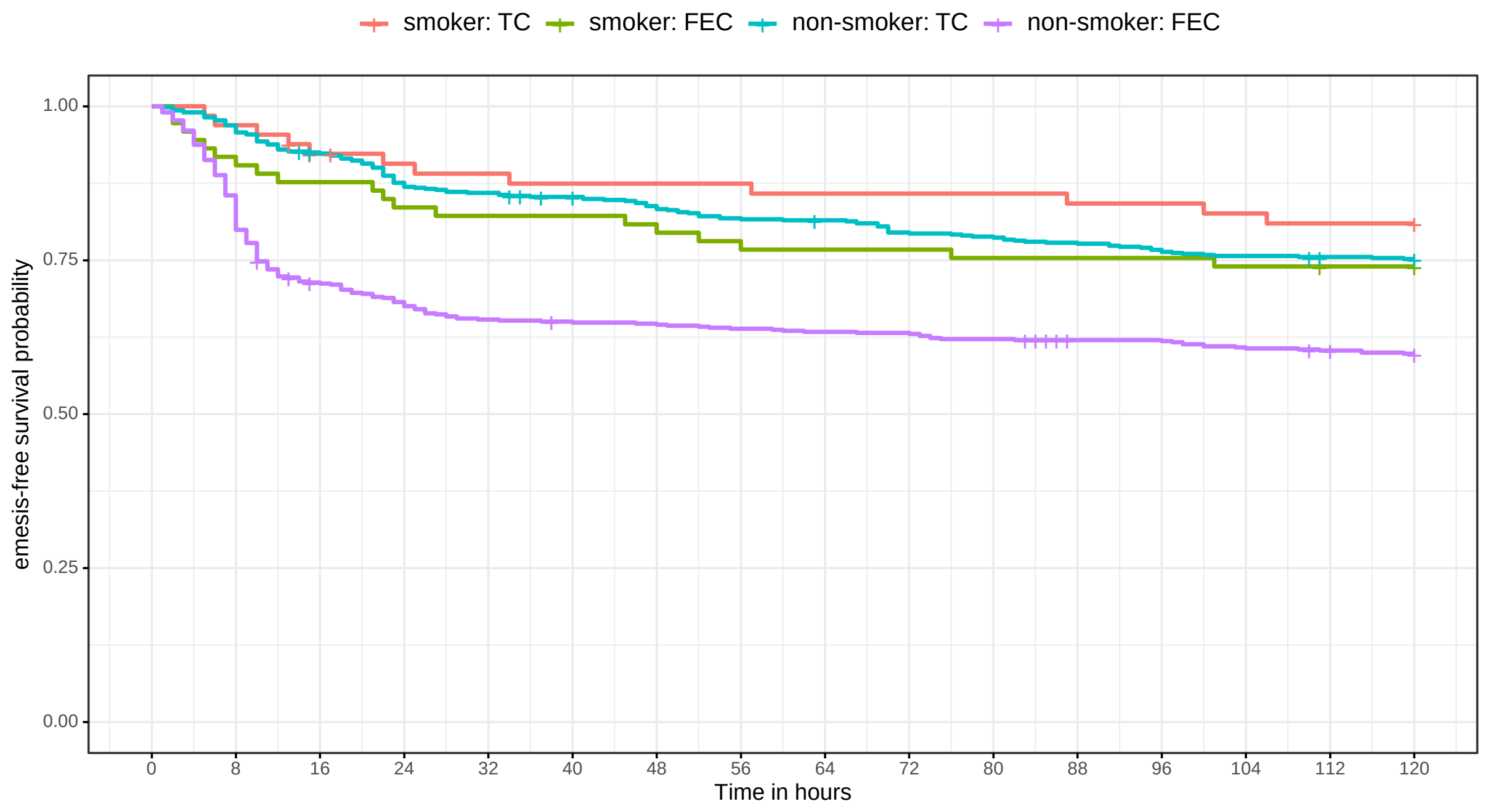

Number at risk

	0	8	16	24	32	40	48	56	64	72	80	88	96	104	112	120
smoker: TC	65	63	58	56	55	54	54	54	53	53	53	52	52	51	50	50
smoker: FEC	73	67	64	61	60	60	59	57	56	56	55	55	55	54	53	53
non-smoker: TC	612	593	564	534	524	517	507	495	492	480	476	470	463	457	454	452
non-smoker: FEC	608	520	428	409	392	389	387	382	379	378	372	366	366	359	355	351

Time in hours

Supplemental figure 7. Emesis-free survival described via Kaplan Meier curves between the FEC and the TC group for alcohol subgroups. A shows cycle 1, B cycle 2, and C cycle 3.

A

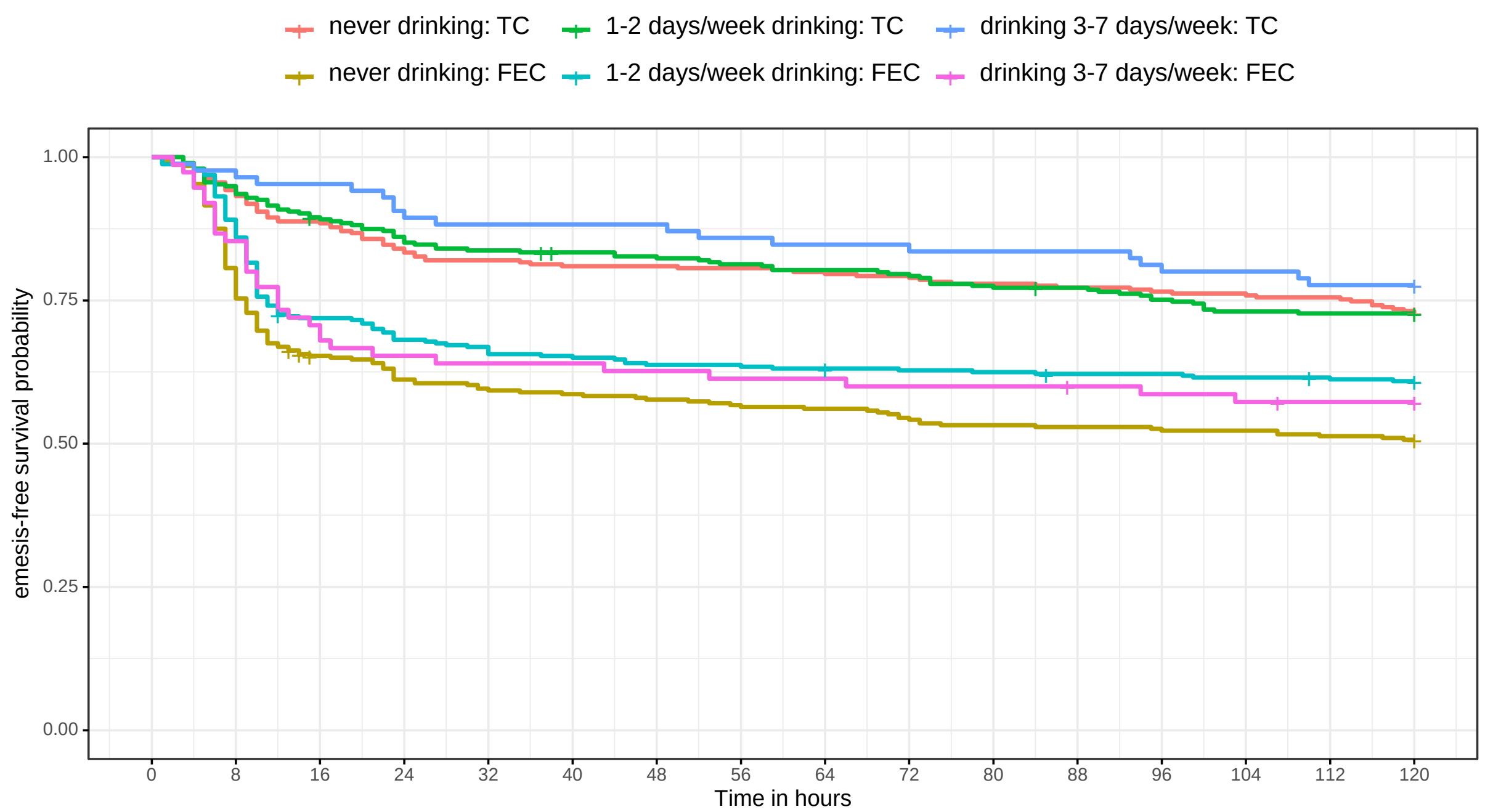

Number at risk

	0	8	16	24	32	40	48	56	64	72	80	88	96	104	112	120
never drinking: TC	294	277	261	247	241	238	238	237	235	233	229	227	225	224	222	215
never drinking: FEC	320	258	205	192	187	184	181	178	176	171	167	166	165	164	161	159
1-2 days/week drinking: TC	295	280	263	253	246	243	241	237	234	232	226	224	218	212	211	211
1-2 days/week drinking: FEC	320	285	229	217	213	208	203	203	201	199	198	196	196	194	193	191
drinking 3-7 days/week: TC	85	83	81	77	75	75	75	73	72	72	71	71	69	68	66	66
drinking 3-7 days/week: FEC	75	64	53	49	48	48	47	46	46	45	45	44	43	42	41	41

Time in hours

B

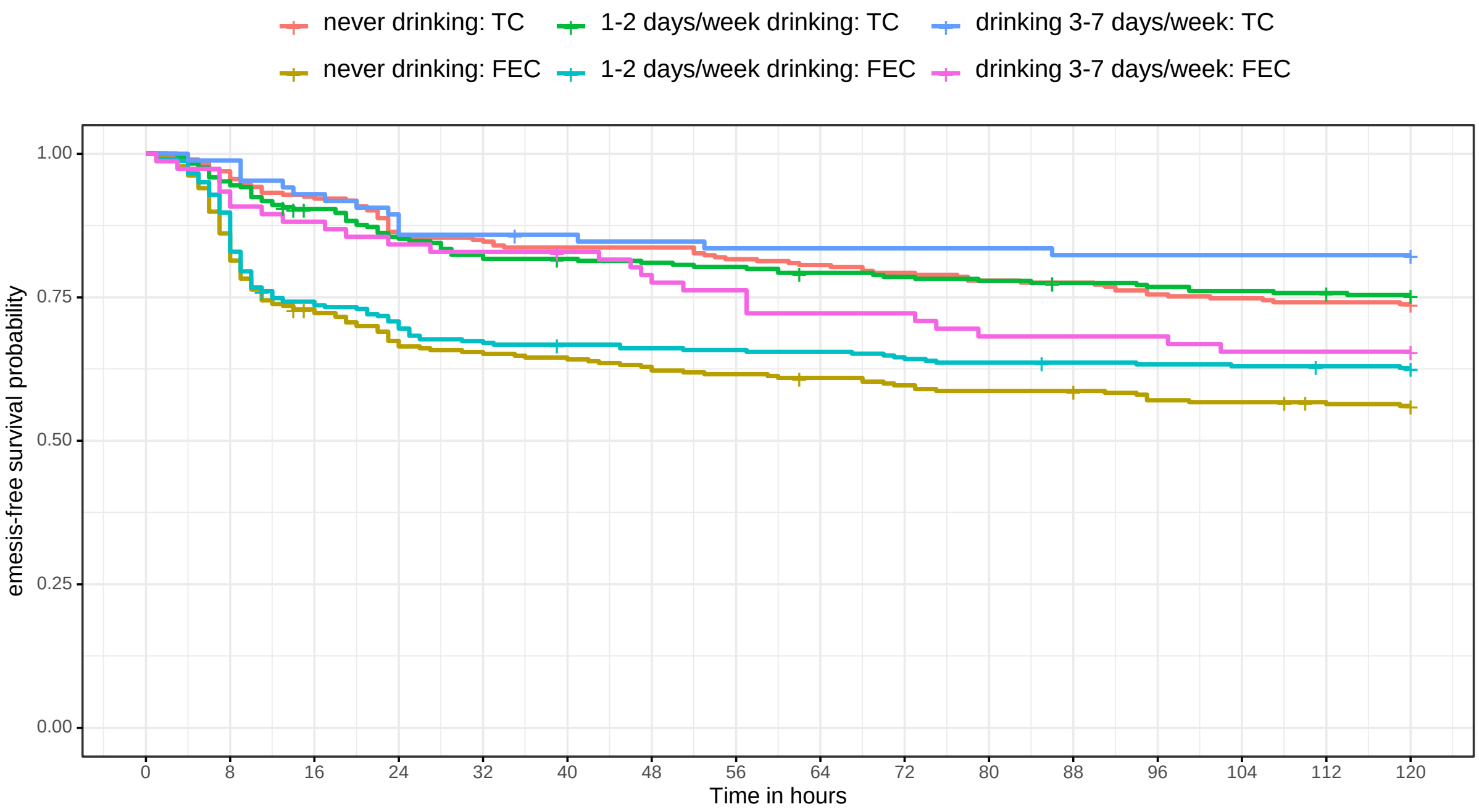

Number at risk

	0	8	16	24	32	40	48	56	64	72	80	88	96	104	112	120
never drinking: TC	294	285	272	254	250	246	246	240	237	233	229	227	221	219	217	216
never drinking: FEC	317	273	226	209	203	200	195	191	188	184	181	181	175	174	172	170
1-2 days/week drinking: TC	291	277	260	246	237	234	232	230	226	224	222	220	218	216	215	213
1-2 days/week drinking: FEC	322	289	238	227	216	213	211	210	209	206	203	202	201	200	199	198
drinking 3-7 days/week: TC	85	84	79	76	73	72	71	70	70	70	70	69	69	69	69	69
drinking 3-7 days/week: FEC	76	71	67	64	63	62	59	57	54	54	51	51	51	49	49	49

Time in hours

C

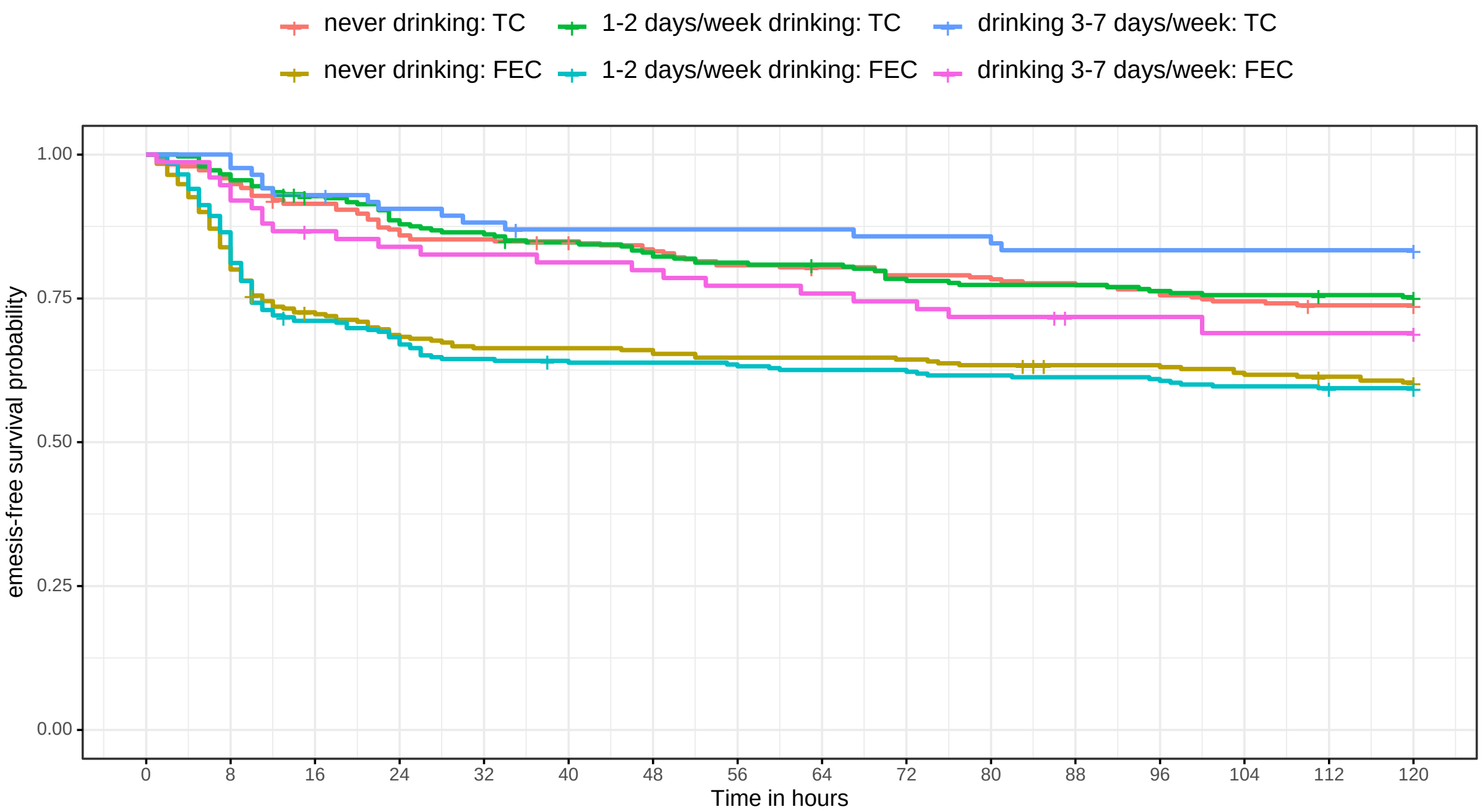

Number at risk

	0	8	16	24	32	40	48	56	64	72	80	88	96	104	112	120
never drinking: TC	292	280	266	253	248	246	241	233	231	227	226	223	219	214	211	211
never drinking: FEC	310	260	221	209	202	202	201	197	197	196	193	190	190	186	183	180
1-2 days/week drinking: TC	290	280	265	253	247	241	236	231	229	222	219	219	216	214	213	212
1-2 days/week drinking: FEC	318	275	225	216	204	202	201	200	197	197	194	193	192	188	187	186
drinking 3-7 days/week: TC	85	85	79	76	74	72	72	72	72	71	71	69	69	69	69	69
drinking 3-7 days/week: FEC	75	71	64	62	61	60	59	57	56	55	53	51	51	49	49	49

Time in hours

Supplemental figure 8. Emesis-free survival described via Kaplan Meier curves between the FEC and the TC group for BMI subgroups. A shows cycle 1, B cycle 2, and C cycle 3.

A

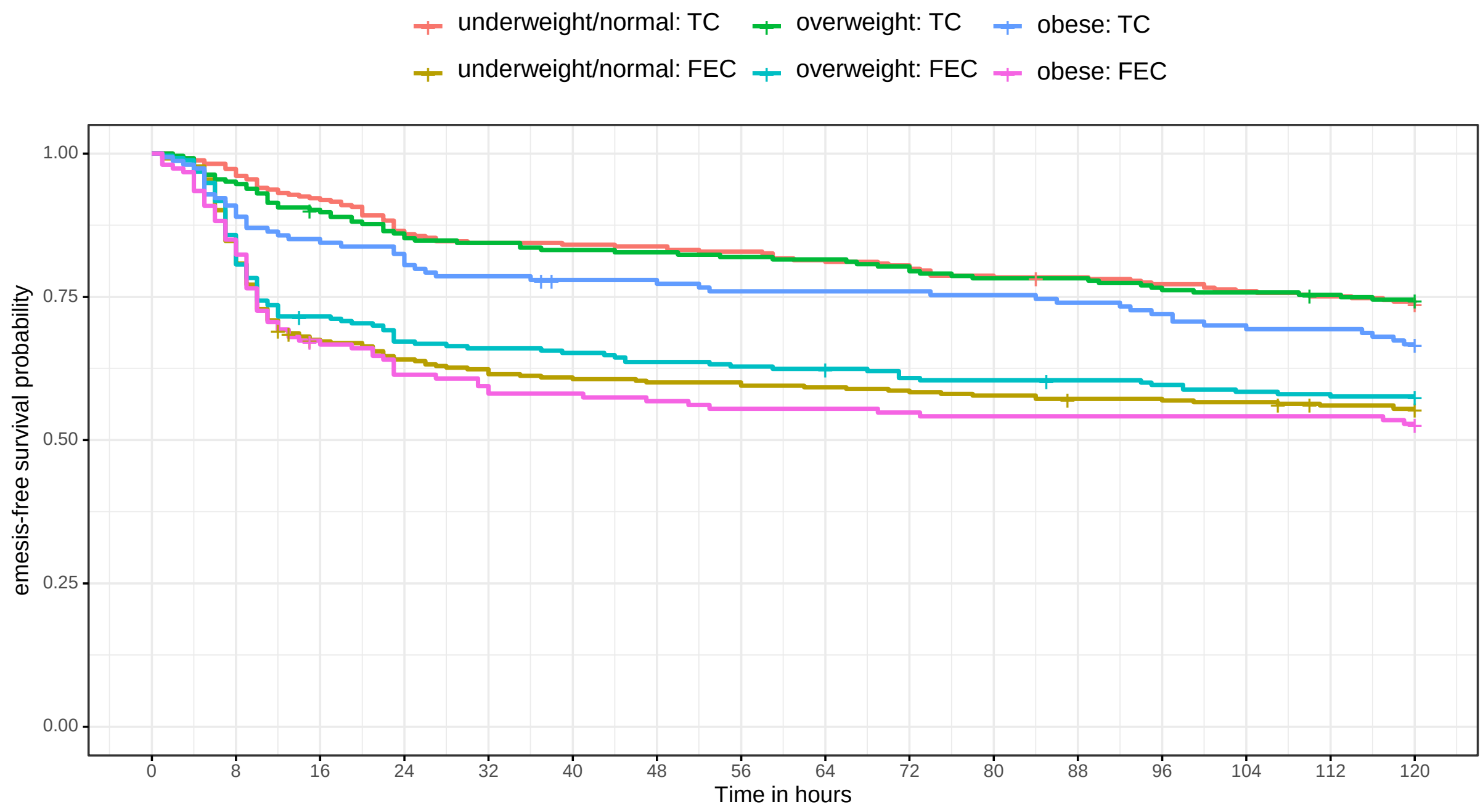

Number at risk

	0	8	16	24	32	40	48	56	64	72	80	88	96	104	112	120
underweight/normal: TC	333	324	307	288	281	280	279	276	271	268	262	260	256	252	249	246
underweight/normal: FEC	354	300	236	224	218	213	210	210	207	205	202	198	198	196	192	190
overweight: TC	244	232	219	209	205	202	201	199	198	195	190	190	186	184	182	180
overweight: FEC	253	217	180	169	166	164	160	158	157	152	151	150	148	145	144	143
obese: TC	154	140	131	127	121	118	118	115	115	115	114	112	109	106	105	101
obese: FEC	153	130	102	93	90	88	86	84	84	83	82	82	82	82	82	80

Time in hours

B

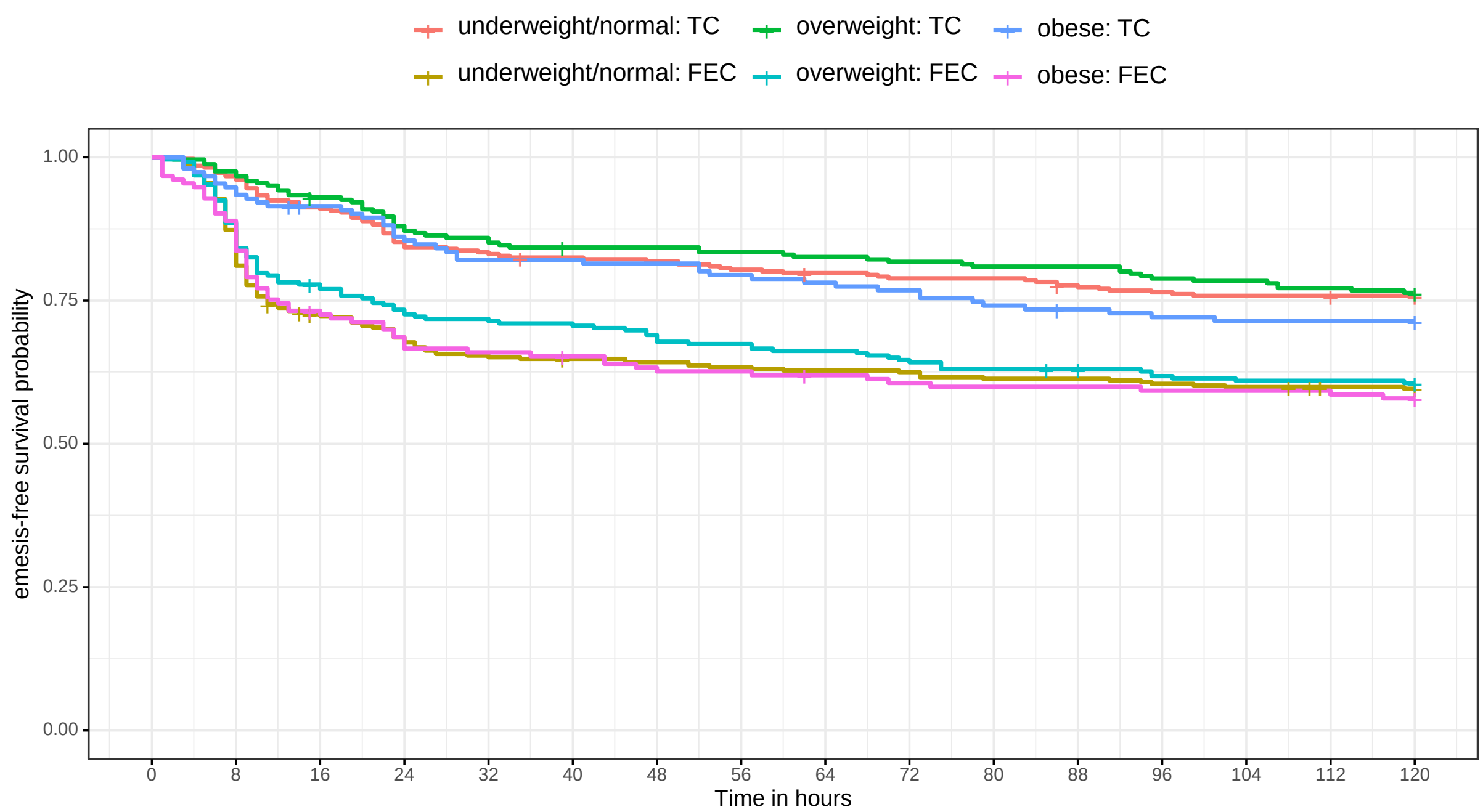

Number at risk

	0	8	16	24	32	40	48	56	64	72	80	88	96	104	112	120
underweight/normal: TC	331	320	302	282	276	272	270	265	262	259	259	254	250	248	248	247
underweight/normal: FEC	354	309	252	238	227	224	222	219	217	216	212	212	209	207	204	203
overweight: TC	242	236	224	212	207	202	202	200	198	196	194	194	189	188	185	183
overweight: FEC	252	223	195	184	180	178	173	169	166	162	158	157	153	151	151	150
obese: TC	152	144	137	129	123	123	122	119	117	115	111	109	107	106	106	106
obese: FEC	153	136	111	104	100	98	95	94	92	90	89	89	88	88	88	86

Time in hours

C

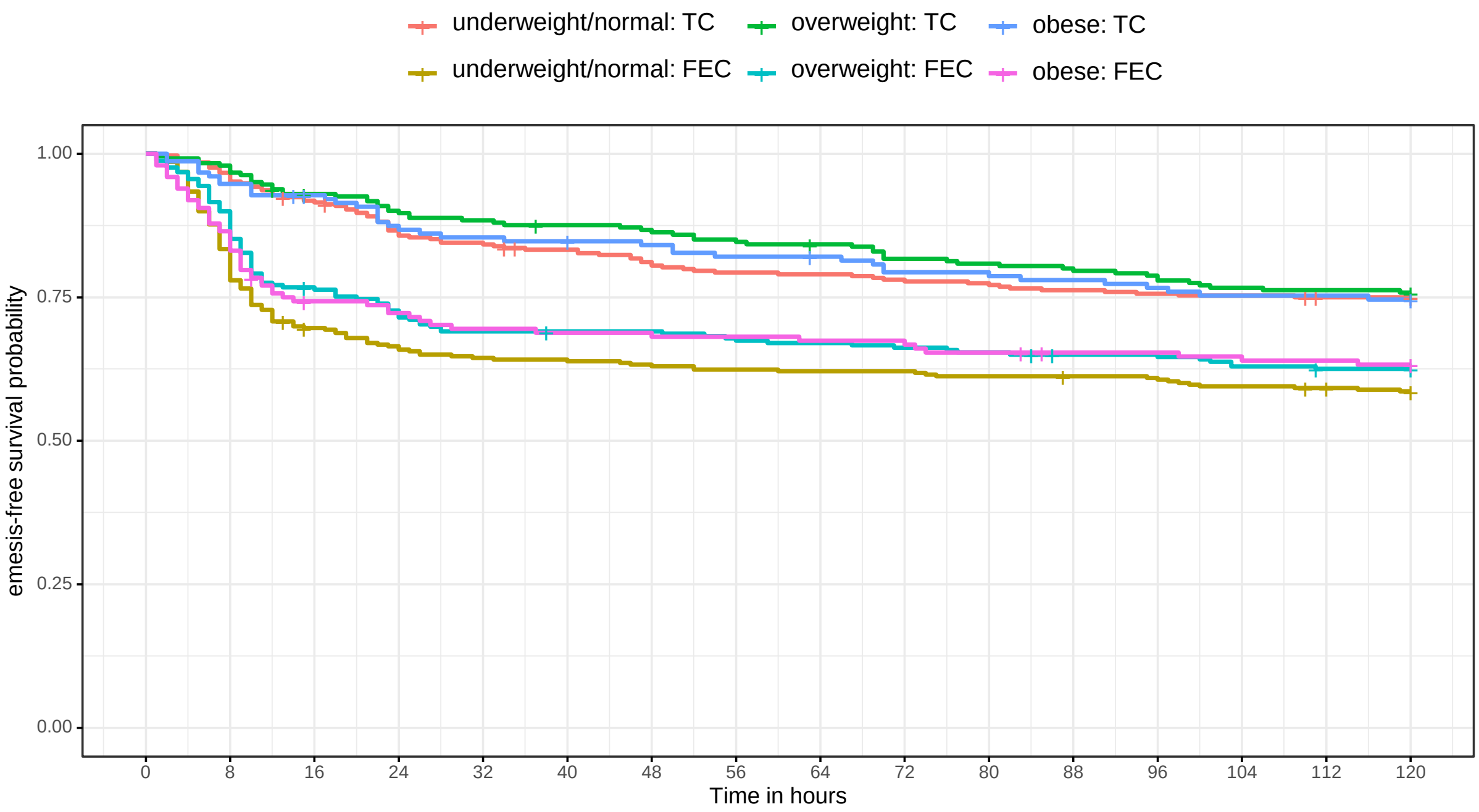

Number at risk

	0	8	16	24	32	40	48	56	64	72	80	88	96	104	112	120
underweight/normal: TC	330	319	302	284	277	271	264	258	257	254	252	248	246	245	242	242
underweight/normal: FEC	349	291	240	229	222	221	218	215	214	214	211	210	209	204	202	199
overweight: TC	242	237	223	216	212	209	207	203	200	194	192	190	187	182	181	180
overweight: FEC	249	224	190	180	171	170	170	167	165	163	161	158	158	153	151	151
obese: TC	152	144	139	131	128	127	125	122	121	117	117	115	113	111	111	110
obese: FEC	148	128	108	105	101	100	100	99	98	98	95	93	93	92	91	90

Time in hours
